# A longitudinal investigation of quality of life and negative emotions in misophonia

**DOI:** 10.3389/fnins.2022.900474

**Published:** 2022-07-22

**Authors:** Bridget Dibb, Sarah E. Golding

**Affiliations:** School of Psychology, Faculty of Health and Medical Sciences, University of Surrey, Guildford, United Kingdom

**Keywords:** misophonia, quality of life, misophonia response scale, anger, disgust, anxiety, depression

## Abstract

**Aims:**

This longitudinal study examined the role of anger, disgust, and anxiety in the experience of misophonia, the quality of life of those with self-reported misophonia in comparison to those without misophonia, and the association of misophonia and quality of life over time.

**Methods:**

An online longitudinal survey was conducted, with misophonia, anger, disgust, anxiety, depression, self-esteem, and quality of life measured at two time points (6-months apart) in two groups of people (those with self-reported misophonia and those without misophonia).

**Results:**

Anger and disgust emerged as the primary predictors of misophonic responses. Anxiety and depression were not significantly associated with misophonia over time. Differences in quality of life were observed between those with and without self-reported misophonia in the current study, with lower scores across the SF-36 domains of role limitations due to emotional problems, energy/fatigue, emotional wellbeing, social functioning, and general health for those with misophonia compared to those without misophonia. Compared with other studies, scores for those with self-reported misophonia were lower than those with long-term physical conditions, similar to those with tinnitus, but higher than those with obsessive compulsive disorder. Misophonia was predictive of quality of life over time but only on two domains: role limitations due to emotional problems (predictors: avoidance, emotional responses, and impact on participation in life) and pain (predictor: impact on participation in life). Depression remained a strong predictor of quality of life over time.

**Conclusion:**

Anger and disgust are more strongly associated with the experience of misophonia than anxiety. Quality of life in people with self-reported misophonia is lower than in the general population and may be similar to those with tinnitus. Depression, avoiding triggers, the extent of the emotional response, and perceived impact on participation in life are associated with perceptions of lower quality of life over time for people with self-reported misophonia.

## Introduction

Misophonia, an aversion to everyday sensory stimuli, is associated with an extreme reaction (emotional and/or physiological) in response to certain trigger stimuli, which are usually auditory stimuli (Jastreboff and Jastreboff, 2001; Edelstein et al., 2013; Schröder et al., 2013; Jager et al., 2020; Swedo et al., 2022). While our knowledge and understanding of the characteristics of misophonia are growing this remains an understudied condition. This study aimed to add to our understanding of misophonia by assessing the association with negative emotions (such as anger, disgust, and anxiety), to determine the quality of life for people with self-reported misophonia using a widely used scale that would facilitate comparisons with other conditions, and to determine the extent to which misophonia is associated with quality of life over time.

Research to date shows that the catalyst that brings about a misophonic response is usually an auditory occurrence, but may sometimes be visual (Swedo et al., 2022). The misophonic response varies between individuals both in terms of the type of trigger, and the intensity and duration of the response (Jastreboff and Jastreboff, 2001; Edelstein et al., 2013; Schröder et al., 2013). The most common auditory triggers are, “mouth-oriented” sounds (for example, chewing and lip smacking), however, visual triggers are also reported; while these are often visual stimuli associated with trigger sounds (for example, seeing someone chewing) (Edelstein et al., 2013; Schröder et al., 2013; Jastreboff and Jastreboff, 2014; Wu et al., 2014; Dozier, 2015; Taylor, 2017; Jager et al., 2020), there are examples of non-mouth visual triggers (for example, leg jiggling and hair twirling) (Jastreboff and Jastreboff, 2001). The source of the trigger also varies with some people being triggered by a person, or people, and others triggered by inanimate sounds (for example, clock ticking, machine noises etc.) (Edelstein et al., 2013). For those who are triggered by a person, some can name specific people while others report being triggered by anyone.

Misophonia is currently an unclassified condition, and there has been an ongoing debate regarding diagnostic criteria and definitions for misophonia (Schröder et al., 2013; Dozier et al., 2017; Taylor, 2017; Jager et al., 2020; Swedo et al., 2022). Indeed, since our study was designed and conducted, a recent consensus statement has been published stating that the predominant triggers for misophonia are auditory, and that there are different dimensions to misophonia including both emotional and physical responses to triggers. However, while many of us find some noises annoying, as yet it is unclear the extent to which this experience differs from those who reportedly have a stronger response, i.e., those with misophonia. A few studies have shown that people recruited from the general population can be classified as having weak or strong responses (for example, Wu et al., 2014; McKay et al., 2018). Both these studies show that responses to auditory sensitivities are also experienced in those who do not have or who are not aware they have misophonia. Both studies also carried out comparison analysis by splitting their group according to a proposed cut off (a score of 7 of the Misophonia Questionnaire (MQ; Wu et al., 2014) and showed significant differences in these groups. For example, the McKay et al. study showed that the group mean for auditory sensitivities for those scoring under 7 (i.e., those who probably don’t have misophonia) was 3.37 and the mean for those scoring over 7 (those the authors suggest may have misophonia) was 8.41, indicating that, if the second group does indeed have misophonia, there is a clear difference in the response to stimuli. This study also reported a significant difference between the “presence of symptoms” subscale (15.42 for those scoring above the cut off and 10.11 for those scoring below) and the “emotions and behaviors” subscale (17.49 for those above the cut off and 9.84 for those below the cut off), again showing a potential difference should the first group have misophonia. The higher mean of the emotions and behavior subscale suggest these domains are more of a concern for those who have a response to triggers. Further research is needed to determine the extent of any differences between those that feel they have misophonia and those who don’t as we lack clear understanding of the extent of the emotional and physiological responses between these two groups. This knowledge will help to clarify the experience of misophonia for the individual.

Understanding more about the response to triggers includes understanding the dominant emotions associated with the condition. The literature shows some conflicting evidence in this regard. Anger, disgust, and anxiety have all been previously reported as important in misophonia, and anxiety-related disorders have been evidenced in several studies. Anxiety was reported in two qualitative studies which recruited misophonia-specific samples (Edelstein et al., 2013; Dozier and Morrison, 2017) and was reported to be associated with the trigger in a cross-sectional survey (Rouw and Erfanian, 2018). In addition, studies which recruited participants from the general population found anxiety to be associated with misophonia (Cusack et al., 2018) and anxiety to be a mediator of the relationship between misophonia and anger (Wu et al., 2014). One study compared anxiety in those who reported higher (versus lower) scores on the Sound Sensitivity subscale (of the MQ; Wu et al., 2014) and found anxiety levels to be significantly greater in those who scored higher for misophonia (McKay et al., 2018). However, some studies, while reporting anxiety, found it was not the main emotion experienced (Schröder et al., 2013). Jager et al. (2020) also reported that anxiety was not the primary response in their study where only 1% of the people with misophonia reported anxiety. These studies show that anxiety is present in people with misophonia, however the lack of consistency in studies that assessed anxiety as either a precursor to misophonia or a consequence means that the evidence base is not clear.

Anger has also been reported in both qualitative and quantitative studies of misophonia (Edelstein et al., 2013; Schröder et al., 2013; Wu et al., 2014; McKay et al., 2018; Rouw and Erfanian, 2018; Jager et al., 2020) and has been shown to be experienced to a larger degree in those who score higher in comparison to those who score lower on the Sound Sensitivity subscale of the MQ (McKay et al., 2018). This suggests there is more consistent evidence for the role of anger in the experience of misophonia. Disgust is another emotion reported by some studies to be associated with misophonia (Edelstein et al., 2013; Schröder et al., 2013; Rouw and Erfanian, 2018); however, one study found that disgust was not associated with misophonia (Jager et al., 2020). Further research is therefore needed to examine this lack of clarity around the emotions that are important in the experience of misophonia, to contribute to our understanding of misophonia and to inform treatment options.

Studies assessing the impact of misophonia are few. One large quantitative study indicated the negative impact of misophonia on quality of life, which was assessed using various measures including the WHOQOLBref, the Manchester Short Assessment Quality of life questionnaire, and the Sheehan Disability scale (Sheehan, 1983; WHOQOL Group, 1998; Priebe et al., 1999; Jager et al., 2020). Other quantitative studies that used the Sheehan Disability Scale (Sheehan, 1983) have also found misophonia severity to correlate significantly and positively with disability in work/school, social and family domains (Wu et al., 2014; Zhou et al., 2017). However, qualitative evidence has shown contradictory reports, where some participants report a large impact and others no impact at all (Edelstein et al., 2013). Research exploring self-reported quality of life in people with misophonia, using a validated and widely used scale such as the Medical Outcomes Study SF-36 (Ware and Sherbourne, 1992), to enable comparison to those with other long-term conditions and those without, will further our understanding of how misophonia impacts life.

This study therefore aimed to help address the current lack of clarity surrounding the role of different negative emotions in misophonia, and to provide insight about the impact of misophonia on quality of life. Specifically, we examined the role of anger, disgust and anxiety in the experience of misophonia, the quality of life of those with self-reported misophonia in comparison to those without misophonia, and the association of misophonia and quality of life over time.

## Materials and methods

### Design

A longitudinal design was used. Survey data were collected online from two samples (those with self-reported misophonia and those without) at two time points, approximately 6 months apart.^1^ Data collection occurred from July–October 2020 (baseline) and January–May 2021 (follow-up). This prospective design allowed measurement of predictor variables at baseline and the outcome variables at follow-up. This design enabled us to answer the main questions examining which factors are associated with the experience of misophonia, how the experiences of people with self-reported misophonia differ from those without, and how quality of life is affected for those with misophonia.

### Participants

Participants with and without self-reported misophonia were recruited to complete the same online survey. To enable recruitment of participants likely to have misophonia, an email advertisement was shared through the Misophonia Institute, United States. Although based in the United States, the Institute is associated with over 5,000 people worldwide, most of whom have self-diagnosed misophonia. To enable recruitment of people without misophonia, the study was also advertised to the general public using social media and snowball sampling. GPower analysis (Faul et al., 2007) confirmed a sample of 114 was needed for each group to achieve a power of > 0.8; however, to account for attrition rates, common in longitudinal research (Boys et al., 2003; Gustavson et al., 2012), we aimed for 400 participants per group at baseline.

### Measures

#### Demographics

At baseline, participants were asked to provide their age, gender, and ethnicity (all optional). They were also asked to “Please indicate below if you have any of the following conditions,” with the response options being *Yes*, *No*, and *Prefer not to say*. The conditions were presented to participants as follows:

1. Tinnitus (ringing in the ears)
2. Misophonia (sensitivity to sensory stimuli)
3. Any other sensory conditions (e.g., hyperacusis, sensory processing disorder, or other sensory disorder)
4. Vertigo (dizziness)
5. Anxiety
6. Depression
7. Any other conditions (please specify).

Self-reported responses to the *misophonia* item in this list were used to classify participants into two groups for analysis; those who responded *yes* were classified as having misophonia (hereafter referred to as the “misophonia group”) and those who responded *no* were classified as not having misophonia (hereafter referred to as the “general population group”). Any participants who responded *Prefer not to say* for this item were excluded from the final analysis, as they could not be classified into either of the two groups. Although there are limitations with asking people to self-identify as to whether they have misophonia or not, given the lack of agreed international diagnostic criteria at the time of our study and the fact that misophonia is not currently a classified condition, we considered this approach to be appropriate for the current study.

At follow-up we asked participants to state their country of residence to indicate geographic spread of the sample. We had omitted this at baseline but included at follow-up as we were collecting data from an international sample.

#### Misophonia triggers

At baseline, those participants who reported they had misophonia were asked an additional question: “Please indicate below which types of stimuli you experience as misophonia triggers (select all that apply).” At the time this study was designed, there was no consensus as to which stimuli are characteristic of misophonia; for this reason we decided not to restrict the response to just visual or auditory triggers so response options included: *Sounds*, *Sights*, *Touch*, *Smells*, *Taste*, *Other (please specify)*.

At follow-up, participants were asked to provide additional information about the sensory domains in which they are triggered; participants were asked to indicate from a pre-specified list which specific types of sound, sight, smell or touch triggered them. Participants were also asked to indicate who, from a pre-specified list, they were triggered by (e.g., parent, grandparent, and romantic partner). The items included in these lists were informed by unpublished data collected from people with self-reported misophonia during a previous study (Dibb et al., 2021).

#### Workplace characteristics

Three items exploring participants’ work situation were also included in the baseline questions. Participants were asked about the environment in which they carry out their core day-to-day activities, i.e., where they spend “most of their time” (including work or studies outside the home and housework). First, participants were asked to select what their **current usual** working environment is from a list of 15 response options, including a range of office-based options, other indoor settings such as retail, health/social care, education, and outdoor settings. The second question asked participants to indicate how many people **usually** share their usual working environment (*None – I usually work on my own*, *1–5*, *6–10*, *11–20*, *21–50*, *50*+). The final work-related question asked participants to rate the **usual** noise level in their usual working environment [with a 7-point Likert scale ranging from 1 (*very little noise*) to 7 (*very noisy*)].

#### Primary outcome variables – misophonic response and quality of life

The response to sensory stimuli (i.e., the misophonic response) was measured using the Misophonia Response Scale (MRS) (Dibb et al., 2021) which consists of 22 items, 19 of which form three subscales [emotional response (seven items), physiological response (seven items), and perceived participation in life (five items)]. The additional three items measure the frequency of a response to a trigger, the degree of avoidance of triggering situations and the perceived time it takes to recover from a response. The MRS is not intended to be used as a diagnostic tool, but it can be used to measure the extent of individuals’ responses to sensory stimuli. For this study, the scale was amended so that the word “misophonia” was omitted; instead items asked about “response to stimuli” to ensure that it made sense to both those with self-reported misophonia and those without. All items were rated on a 7-point scale. A total score and a weighted score can also be calculated. Internal consistency for all three MRS subscales was good at both baseline and follow-up, ranging from 0.77 to 0.96 (Table 1).

**TABLE 1:** Cronbach’s alpha for whole sample and by group at baseline and follow-up.

Measure	Baseline			Follow-up		
	Whole sample (*n* = 994)	Misophonia (*n* = 491)	General population (*n* = 503)	Whole sample (*n* = 222)	Misophonia (*n* = 127)	General population (*n* = 95)
**SF-36**						
Physical functioning	0.90	0.91	0.87	0.90	0.90	0.88
Role limitations physical health	0.88	0.89	0.85	0.87	0.87	0.87
Role limitations emotional problems	0.85	0.85	0.82	0.81	0.82	0.76
Energy/Fatigue	0.87	0.85	0.87	0.87	0.85	0.88
Emotional wellbeing	0.87	0.84	0.86	0.86	0.83	0.85
Social functioning	0.86	0.82	0.85	0.85	0.85	0.79
Pain	0.86	0.88	0.83	0.85	0.85	0.86
General health	0.82	0.83	0.80	0.82	0.80	0.84
**Psychosocial variables**						
Anger	0.79	0.77	0.69	0.76	0.73	0.70
Disgust	0.88	0.87	0.80	0.85	0.82	0.80
Anxiety	0.90	0.88	0.88	0.89	0.87	0.90
Self-esteem	0.93	0.92	0.91	0.93	0.93	0.91
Depression	0.87	0.84	0.86	0.87	0.84	0.87
**MRS subscales**						
Emotional	0.96	0.85	0.91	0.95	0.87	0.91
Physiological	0.91	0.86	0.90	0.90	0.89	0.86
Participation	0.90	0.78	0.89	0.89	0.77	0.90

Quality of life was measured with the Medical Outcomes Study SF-36 health survey (Ware and Sherbourne, 1992), sourced from RAND Corporation.^2^ This is a 36-item multidimensional quality of life questionnaire which measures health on eight dimensions (physical functioning, role limitations due to physical health, role limitations due to emotional problems, energy/fatigue, emotional wellbeing, social functioning, pain, and general health). This validated questionnaire has been used widely and developed to be used across many different conditions for the purpose of comparison (Bowling, 2005). It has been used in physical (e.g., Scharloo et al., 2007), neurological (e.g., Baca et al., 2015), and mental health conditions (e.g., Friedman et al., 2005). It was chosen specifically because it is so widely used, which enables us to show the impact of misophonia on quality of life in comparison to quality of life in other conditions reported in the literature. Internal consistency for all eight SF-36 subscales was good at both baseline and follow-up, ranging from 0.76 to 0.91 (Table 1).

#### Predictor variables – psychosocial constructs

Three scales measuring negative emotions (anger, disgust, and anxiety) were included to determine the association of these emotions with the experience of misophonia and to determine differences between people with self-reported misophonia and those without. Anger was measured with the 6-item Brief Anger and Aggression Questionnaire (Maiuro et al., 1987), with all items measured on a 5-point Likert scale (*extremely unlikely* to *very likely*). Disgust was measured with the 6-item Propensity subscale of the revised Disgust Propensity and Sensitivity scale (Fergus and Valentiner, 2009), with all items measured on a 5-point Likert scale (*never* to *always*). Anxiety was measured with the 6-item State Trait Anxiety Inventory (Marteau and Bekker, 2020), rated on a 4-point scale (*not at all* to *very much*). Higher total scores on each scale indicate greater levels of anger, disgust, or anxiety.

Depressive symptoms [which show a strong association with quality of life (Patrick et al., 2000; Schram et al., 2009; Kim et al., 2018) and misophonia (McKay et al., 2018; Quek et al., 2018; Alekri and Al Saif, 2019)] and self-esteem (which shows a strong association with quality of life; Porter and Boothroyd, 2015; Teoh et al., 2015; Huang et al., 2018) were also measured to account for their effects. Depression was measured with the CES-D-10 scale (Radloff, 1977; Andresen et al., 1994) with consists of 10 items all rated on a 4-point scale (*rarely or none of the time* to *all of the time*). A high score indicates more depressive symptoms. Self-esteem was measured using Rosenberg’s Self-Esteem Scale (Rosenberg, 1965), which consists of 10 items all rated on a 4-point scale (*strongly agree* to *strongly disagree*). The scale was scored so that a high score indicates high self-esteem.

Hereafter, these five variables (anger, disgust, anxiety, self-esteem, and depression) are collectively referred to as the *psychosocial variables*. Internal consistency for these psychosocial variables was good at both baseline and follow-up, ranging from 0.69 to 0.93 (Table 1).

### Procedure

After ethical approval was received from the University of Surrey Ethics Committee, the Misophonia Institute, United States, advertised the online study via their social media channels and via email to those associated with the Institute at the time. The advert was also shared more widely on social media by both authors and additional participants were recruited through snowball sampling. Informed consent to participate was gained at the time of completing the online survey. At baseline, participants first completed measures of demographical information (age, gender, ethnicity, co-morbid conditions, and work environment), then completed the measures of quality of life, anxiety, depression, anger, disgust, self-esteem, and finally responses to sensory stimuli. Participants were also asked to provide their email address if they were willing to be contacted 6 months later with the follow-up questionnaire; those who agreed were emailed a link to the follow-up questionnaire (which consisted of the same predictor and outcome variable measures as at baseline but also included the question on country of residence).

### Analysis

Descriptive statistics were used to determine the sample characteristics. Independent *t*-tests were used to examine differences between the two groups (those with and without self-reported misophonia), at both baseline and follow-up, with regard to the response to sensory stimuli (MRS scores), levels of anger, disgust, anxiety, self-esteem, depression, and perceptions of quality of life. Paired-samples *t*-tests were used to examine any changes in these variables within-participants over time.^3^ As multiple inferential tests were performed on the same outcome variables, the *p*-values for determining statistical significance were adjusted using the Bonferroni correction (*p* = 0.05/number of tests) (Tabachnick and Fidell, 2007). For the SF-36 and MRS outcomes the adjusted *p* = 0.0167 (0.05/3), and for the psychosocial outcomes the adjusted *p* = 0.025 (0.05/2).

Hierarchical multiple regressions were conducted to explore predictors of the misophonic response and of quality of life in people with self-reported misophonia, both cross-sectionally and longitudinally. To achieve more clarity on how anger, disgust, and anxiety relate to the misophonic response, they were entered in a separate block to depression (McKay et al., 2018; Quek et al., 2018; Alekri and Al Saif, 2019), as we wanted to be able to account for the effects of anger, disgust and anxiety without the influence of depression (which has been shown to have a strong association with misophonia). To achieve this, predictor variables were entered as follows: (block one) age, (block two) anger, disgust, anxiety, and (block three) depression. Eight regressions were conducted to predict eight misophonic outcomes: frequency, recovery, avoidance, emotional responses, physiological responses, participation in life, severity MRS scores, and weighted MRS scores. To predict perceived quality of life, predictor variables were entered as follows: (block one) age, (block two) MRS scores for frequency, recovery, avoidance, emotional responses, physiological responses, and participation, and (block three) anxiety, self-esteem, and depression. Eight regressions were conducted to predict each of the eight SF-36 sub-scales: physical functioning, role limitations due to physical health, role limitations due to emotional problems, energy/fatigue, emotional wellbeing, social functioning, pain, and general health. All models were bootstrapped.^4^ For brevity of reporting, only summary statistics are reported for the regression models; full details of the regression models are available in the Appendix.

## Results

### Participant demographics

The baseline survey was started 1,433 times, but 439 responses were deleted during data screening for the following reasons: aged under 18 (*n* = 6), answered prefer not to say to misophonia grouping question (*n* = 3), likely a duplicate response based on emails provided for follow-up (*n* = 6), did not progress beyond demographics (*n* = 266) or complete all measures (*n* = 158). This left a total of 994 participants (77.3% female) who completed the survey at baseline. Participants were aged 18–91 years (mean (*m*) = 47.23, standard deviation (*SD*) = 14.54), and most (90.9%) reported their ethnicity as White. Of these 994 participants, 491 stated they did have misophonia (the misophonia group) and 503 stated they did not have misophonia (the general population group). For full demographic details see Table 2.

**TABLE 2:** Participants’ demographic characteristics.

Characteristic	Baseline					Follow-up				
	Whole sample (*n* = 994)	Misophonia (*n* = 491)	General population (*n* = 503)	Pearson’s χ^2^ (df)	*p*	Whole sample (*n* = 222)	Misophonia (*n* = 127)	General population (*n* = 95)	Pearson’s χ^2^ (df)	*p*
**Gender**				35.62 (3)	<0.001				11.05 (2)	0.004
Male	215 (21.6%)	68 (13.8%)	147 (29.2%)			49 (22.1%)	18 (14.2%)	31 (32.6%)		
Female	768 (77.3%)	416 (84.7%)	352 (70.0%)			169 (76.1%)	107 (84.3%)	62 (65.3%)		
Other	10 (1.0%)	6 (1.2%)	4 (0.8%)			4 (1.8%)	2 (1.6%)	2 (2.1%)		
Prefer not to say	1 (0.1%)	1 (0.2%)	0 (0.0%)			0 (0.0%)	0 (0.0%)	0 (0.0%)		
**Ethnicity**				2.56 (5)	0.77				3.30 (3)	0.35
Asian	21 (2.1%)	9 (1.8%)	12 (2.4%)			0 (0.0%)	0 (0.0%)	0 (0.0%)		
Black	9 (0.9%)	4 (0.8%)	5 (1.0%)			1 (0.5%)	1 (0.8%)	0 (0.0%)		
Hispanic/Latina	26 (2.6%)	15 (3.1%)	11 (2.2%)			6 (2.7%)	5 (3.9%)	1 (1.1%)		
White	904 (90.9%)	444 (90.4%)	460 (91.5%)			205 (92.3%)	114 (89.8%)	91 (95.8%)		
Other	33 (3.3%)	18 (3.7%)	15 (3.0%)			10 (4.5%)	7 (5.5%)	3 (3.2%)		
Prefer not to say	1 (0.1%)	1 (0.2%)	0 (0.0%)			0 (0.0%)	0 (0.0%)	0 (0.0%)		
Health conditions*										
Tinnitus	260 (26.2%)	146 (29.7%)	114 (22.7%)	8.69 (2)	0.013	55 (24.8%)	38 (29.9%)	17 (17.9%)	4.22 (1)	0.040
Vertigo	148 (14.9%)	94 (19.1%)	54 (10.7%)	14.99 (2)	0.001	33 (14.9%)	25 (19.7%)	8 (8.4%)	5.45 (1)	0.020
Anxiety	509 (51.2%)	346 (70.5%)	163 (32.4%)	146 (2)	<0.001	108(48.6%)	83 (65.4%)	25 (26.3%)	33.15 (1)	<0.001
Depression	386 (38.8%)	266 (54.2%)	120 (23.9%)	99.37 (2)	<0.001	89 (40.1%)	70 (55.1%)	19 (20.0%)	29.25 (2)	<0.001
Other sensory conditions	114 (11.5%)	105 (21.4%)	9 (1.8%)	101.58 (2)	<0.001	35 (15.8%)	34 (26.8%)	1 (1.1%)	29.16 (2)	<0.001
Any other health conditions	230 (23.1%)	136 (27.7%)	94 (18.7%)	19.10 (2)	<0.001	61 (27.5%)	42 (33.1%)	19 (20.0%)	4.67 (2)	0.097

df, degrees of freedom. *Number of participants who stated they do have this health condition.

An independent *t*-test was conducted to examine group differences at baseline in age, and Pearson’s chi-square tests were conducted to examine group differences in gender, ethnicity, and having other health conditions. Except for ethnicity, there were statistically significant differences across all demographic variables at baseline between the two groups. The misophonia group were on average younger than the general population group [misophonia age range = 18–80, *m* = 44.32, *SD* = 14.54; general population age range = 18–91, *m* = 50.06, *SD* = 13.99; *t*(988.17) = 6.35, *p* < 0.001, *d* = 0.40]. A greater proportion of the misophonia group were female compared with the general population group. For each of the health conditions assessed, participants in the self-reported misophonia group were more likely to report having that health condition than those in the general population group (Table 2).

From the 994 useable baseline responses, 958 participants provided an email at baseline and were invited 6 months later to complete the follow-up survey, which was accessed 246 times. At follow-up, 24 responses were deleted during data screening for the following reasons: aged under 18 (*n* = 3), likely a duplicate response based on emails provided for follow-up (*n* = 6), did not complete all measures (*n* = 11), response could not be matched to baseline as email check provided at follow-up did not match (*n* = 4). Therefore, a total of 222 people participated at follow-up (misophonia, *n* = 127; general population *n* = 95; follow-up rate of 22.33%). Of these 222 participants, 76.1% were female, 92.3% were White, and their age range was 18–80 (*m* = 49.02, *SD* = 15.07). There were no statistically significant differences in ethnicity and age at follow-up, although the misophonia group were on average younger than the general population group [misophonia age range = 18–75, *m* = 47.36, *SD* = 15.04; general population age range = 18–80, *m* = 51.24, *SD* = 14.90; *t*(220) = 1.91, *p* = 0.058, *d* = 0.26]. There were, however, statistically significant differences in gender and health conditions between the two groups at follow-up (Table 2). At follow-up, 221 participants provided their country of residence; most resided in the United States (*n* = 108) and United Kingdom (*n* = 69), followed by Australia (*n* = 11), Canada (*n* = 10), and New Zealand (*n* = 8), with remaining participants residing in 15 other countries (*n* = 1 each).^5^

### Misophonia triggers

At baseline, participants who stated they did have misophonia were presented with an additional question to assess which type of stimuli they experienced as misophonic triggers (Table 3). Sounds were experienced as triggers by all but one participant, and sights were experienced as triggers by 63%. Touch and smells were experienced by around one-quarter of participants, and only a small number of participants reported experiencing taste or other stimuli as triggers.

**TABLE 3:** Number of people with misophonia who report experiencing specific characteristics of triggers or being triggered by particular people.

Characteristic	Baseline (*n* = 490)	Follow-up (*n* = 126)
**Trigger stimulus**		
Sounds	489 (99.8%)	126 (100.0%)
Sights	308 (62.9%)	82 (65.1%)
Touch	131 (26.7%)	35 (27.8%)
Smells	121 (24.7%)	34 (27.0%)
Taste	24 (4.9%)	8 (6.3%)
Other stimuli	26 (5.3%)	5 (4.0%)
**Sound triggers**		
Eating-related mouth sounds	–	114 (89.8%)
Non-eating related mouth sounds	–	101 (79.5%)
Nose sounds	–	105 (82.7%)
Human voice sounds	–	76 (59.8%)
Speech-related sounds	–	48 (37.8%)
Hands or feet sounds	–	85 (66.9%)
Animal-related sounds	–	56 (44.1%)
People’s food-related behavior sounds	–	85 (66.9)
People’s non-food-related behavior sounds	–	86 (67.7%)
Mechanical/digital sounds	–	68 (53.5%)
Other sounds	–	32 (25.2%)
N/A – not triggered by sounds	–	1 (0.8%)
**Sight triggers**		
Mouth-related movement	–	83 (65.4%)
Hand/leg movement	–	70 (55.1%)
Unwashed body part	–	27 (21.3%)
Other sights	–	27 (21.3%)
N/A – not triggered by sights	–	20 (15.7%)
**Smell triggers**		
Perfume smells	–	35 (27.6%)
Chemical smells	–	31 (24.4%)
Minty smells	–	13 (10.2%)
Food or drink smells	–	27 (21.3%)
Rotting or moldy smells	–	33 (26.0%)
Smoke smells	–	29 (22.8%)
Body-related smells	–	49 (38.6%)
Other smells	–	8 (6.3%)
N/A – not triggered by smells	–	55 (43.3%)
**Touch triggers**		
Skin	–	36 (28.3%)
Vibration	–	34 (26.8%)
Other touch	–	14 (11.0%)
N/A – not triggered by touch	–	73 (57.5%)
**Trigger person**		
Parents	–	76 (59.8%)
Grandparents	–	19 (15.0%)
Siblings	–	48 (37.8%)
Children	–	57 (44.9%)
Romantic partner	–	79 (62.2%)
Other family	–	25 (19.7%)
Friends	–	53 (41.7%)
Strangers	–	78 (61.4%)
Neighbors	–	49 (38.6%)
Workplace people	–	69 (54.3%)
Pets/animals	–	34 (26.8%)
Housemates	–	30 (23.6%)
Anyone	–	89 (70.1%)
Myself	–	18 (14.2%)
Other types of people	–	7 (5.5%)
N/A – triggers not related to people	–	2 (1.6%)

*Sample sizes differ from those in other analyses as one participant with misophonia did not complete these questions.

At follow-up, participants with self-reported misophonia were asked to provide more specific information about the types of triggers they experienced, as well as indicating who they experienced being a “trigger person” (Table 3). All but one person with misophonia reported being triggered by sounds and it is evident from participants’ responses that people experience auditory triggers across a variety of different sounds; triggers are clearly not restricted to only one or two types of sound. Common auditory triggers included various types of sounds related to the human body, as well as animal-related sounds and mechanical or digital sounds. Experiencing visual triggers was also reported by most participants; only 15.7% reported that they did not experience any visual triggers. The most common triggering sight was mouth-related movement (65.4%). The proportion of people who reported being triggered by specific types of smell or touch was lower than for specific types of sounds or sights. Nonetheless, at follow-up, 27.0% reported being triggered by smells and 27.8% reported being triggered by touch. In terms of who they experienced as being the source of triggers, people with self-reported misophonia most commonly reported being triggered by anyone (70.1%), romantic partners (62.2%), strangers (61.4%) and parents (59.8%). Only two participants (1.6%) reported not being triggered by people.

### Workplace characteristics

Across the whole sample, and by group, most participants described their current usual working environment during the baseline survey as being at home, followed by being office-based (Table 4). After homes and offices, the next most common workplaces among participants were educational and healthcare settings. Most participants also reported that they usually worked alone or with between 1 and 5 other people usually present in their workplace. There were no statistically significant differences between the two groups in terms of the number of people usually present in their workplace [χ*^2^*(5) = 5.40, *p* = 0.37].

**TABLE 4:** Participants’ workplace characteristics.

Characteristic	Whole sample (*n* = 988)^†^	Misophonia (*n* = 489)	General Population (*n* = 499)
**Number of other people who usually share workplace**			
None – I usually work alone	330 (33.2%)	153 (31.2%)	177 (35.2%)
1–5	408 (41.0%)	200 (40.7%)	208 (41.4%)
6–10	90 (9.1%)	45 (9.2%)	45 (8.9%)
11–20	70 (7.0%)	38 (7.7%)	32 (6.4%)
21–50	49 (4.9%)	30 (6.1%)	19 (3.8%)
50+	41 (4.1%)	23 (4.7%)	18 (3.6%)
**Current usual working environment**			
At home	517 (52.0%)	231 (47.0%)	286 (56.9%)
In a private office at a workplace	61 (6.1%)	29 (5.9%)	32 (6.4%)
In a shared office at a workplace	62 (6.2%)	39 (7.9%)	23 (4.6%)
In an open-place office at a workplace	67 (6.7%)	45 (9.2%)	22 (4.4%)
In a retail outlet	19 (1.9%)	9 (1.8%)	10 (2.0%)
In a café, restaurant or similar environment	12 (1.2%)	7 (1.4%)	5 (1.0%)
In a leisure environment, such as a sports center, bowling alley or similar	4 (0.4%)	2 (0.4%)	2 (0.4%)
In an environment such as a museum, library or similar	2 (0.2%)	2 (0.4%)	0 (0.0%)
In a school or other teaching environment	69 (6.9%)	40 (8.1%)	29 (5.8%)
In health or social care provision	70 (7.0%)	31 (6.3%)	39 (7.8%)
In an emergency services role	3 (0.3%)	1 (0.2%)	3 (0.6%)
In a factory or manufacturing plant	8 (0.8%)	4 (0.8%)	7 (1.4%)
In a motor vehicle (e.g., taxi driver, truck driver, delivery driver, refuse collector)	4 (0.4%)	4 (0.8%)	0 (0.0%)
In natural outdoor environments, such as forests, beaches, gardens, golf courses, farmland	14 (1.4%)	7 (1.4%)	7 (1.4%)
In urban outdoor environments, such as construction sites, road maintenance	6 (0.6%)	2 (0.4%)	4 (0.8%)
Other (please describe)	34 (3.4%)	21 (4.3%)	12 (2.6%)
Retired*	26 (2.6%)	11 (2.2%)	15 (3.0%)
Unemployed*	10 (1.0%)	8 (1.6%)	2 (0.4%)

*Retired and unemployed were not presented as options in the questionnaire, but participants who included this detail under other were re-categorized accordingly.

^†^Six participants did not complete the workplace questions, so results reported in this section are for 988 participants.

Across the whole sample, perceptions of the usual noise level in participants’ working environment were considered just below moderate, i.e., just below the mid-point on the 7-point response scale (*m* = 3.07, *SD* = 1.72). There were, however, significant differences between the two groups, with those in the misophonia group perceiving their working environment as noisier, compared to the general population group [misophonia *m* = 3.53, *SD* = 1.78; general population *m* = 2.61, *SD* = 1.53; *t*(959.27) = 8.75, *p* < 0.001, *d* = 0.56]. Among the whole sample, participants’ perceptions about the usual noise level in their working environment was significantly positively correlated with the number of people with whom they usually share their working environment (*r* = 0.53, *p* < 0.001). The same pattern was seen in the misophonia group (*n* = 489, *r* = 0.49, *p* < 0.001) and the general population group (*n* = 499, *r* = 0.58, *p* < 0.001). Across all participants, those who usually shared their working environment with a greater number of people on average perceived their working environment to be noisier.

### Misophonic responses: Differences between and within participants

Descriptive statistics for MRS scores at baseline and follow-up for those with and without self-reported misophonia are reported in Tables 5–7. Bivariate correlations between the MRS, the psychosocial variables, and quality of life at baseline for those with self-reported misophonia are presented in Table 8. All correlations show an association in the expected direction where a higher misophonia score is associated with more anger, anxiety, disgust and depression, but reduce quality of life and self-esteem. The correlations are significant for all but two of the variables: avoidance does not correlate significantly with anxiety, self-esteem and general health, and the emotional response to triggers does not correlate significantly with physical functioning, physical role limitations, pain, and general health.

**TABLE 5:** Descriptive statistics and between-group tests of difference for misophonic responses, psychosocial variables, and quality of life at baseline.

Measure	Misophonia (*n* = 491)				General population (*n* = 503)				Tests of difference between groups at baseline		
	Item mean (*SD*)	Lower CI, upper CI	Total mean (SD)	Lower CI, upper CI	Item mean (*SD*)	Lower CI, upper CI	Total mean (*SD*)	Lower CI, upper CI	*t*(df)	*p*	Cohen’s *d*
**MRS stand-alone items**											
Frequency	3.98 (1.29)	3.88, 4.09	N/A	N/A	1.52 (0.89)	1.45, 1.60	N/A	N/A	35.04 (867.94)	<0.001	2.23
Recovery	4.05 (1.34)	3.94, 4.16	N/A	N/A	2.35 (1.54)	2.22, 2.49	N/A	N/A	18.65 (978.85)	<0.001	1.18
Avoidance	5.47 (1.64)	5.33, 5.60	N/A	N/A	2.17 (1.67)	2.03, 2.31	N/A	N/A	31.44 (991.92)	<0.001	1.99
**MRS subscales**											
Emotional	5.64 (1.13)	5.54, 5.75	39.49 (7.94)	38.78, 40.23	2.22 (1.19)	2.11, 2.32	15.52 (8.33)	14.78, 16.23	46.46 (991.42)	<0.001	2.95
Physiological	3.24 (1.38)	3.12, 3.36	22.67 (9.64)	21.83, 23.50	1.58 (0.90)	1.52, 1.66	11.08 (6.27)	10.61, 11.64	22.41 (839.09)	<0.001	1.43
Participation	4.16 (1.46)	4.04, 4.28	20.80 (7.27)	20.19, 21.42	1.56 (0.94)	1.48, 1.65	7.79 (4.70)	7.42, 8.23	33.39 (835.45)	<0.001	2.13
**MRS summed scores**											
Severity score	4.37 (1.06)	4.28, 4.45	82.96 (20.16)	81.31, 84.63	1.81 (0.89)	1.74, 1.89	34.40 (16.87)	32.98, 35.84	41.15 (953.85)	<0.001	2.62
Weighted score	20.37 (8.03)	19.71, 21.04	N/A	N/A	4.42 (4.85)	4.01, 4.86	N/A	N/A	37.77 (801.51)	<0.001	2.41
**Psychosocial variables**											
Anger	2.44 (0.73)	2.37, 2.50	14.61 (4.39)	14.23, 14.99	1.82 (0.51)	1.77, 1.87	10.92 (3.05)	10.65, 11.20	15.37 (871.72)	<0.001	0.98
Disgust	3.18 (0.76)	3.11, 3.25	19.07 (4.55)	18.66, 19.49	2.45 (0.56)	2.40, 2.51	14.72 (3.36)	14.39, 15.06	17.10 (901.97)	<0.001	1.09
Anxiety	2.39 (0.74)	2.31, 2.45	14.31 (4.45)	13.88, 14.73	1.82 (0.66)	1.76, 1.88	10.91 (3.99)	10.58, 11.27	12.68 (974.76)	<0.001	0.81
Self-esteem	2.75 (0.64)	2.70, 2.80	27.53 (6.42)	27.04, 28.00	3.24 (0.57)	3.18, 3.29	32.37 (5.71)	31.82, 32.86	12.54 (972.64)	<0.001	0.80
Depression	2.27 (0.63)	2.21, 2.34	22.74 (6.30)	22.15, 23.36	1.79 (0.58)	1.73, 1.84	17.86 (5.79)	17.35, 18.38	12.72 (980.54)	<0.001	0.81
**SF-36**											
Physical functioning	N/A	N/A	84.77 (20.46)	82.98, 86.61	N/A	N/A	88.15 (15.96)	86.70, 89.43	2.91 (926.08)	0.004	0.19
Role limitations – physical health	N/A	N/A	73.57 (37.90)	70.42, 77.09	N/A	N/A	85.24 (29.43)	82.66, 87.52	5.41 (924.03)	<0.001	0.34
Role limitations – emotional problems	N/A	N/A	49.90 (43.40)	46.02, 53.57	N/A	N/A	74.29 (37.07)	71.17, 77.61	9.52 (960.74)	<0.001	0.61
Energy/Fatigue	N/A	N/A	41.47 (21.56)	39.51, 43.55	N/A	N/A	56.47 (21.31)	54.48, 58.27	11.04 (992)	<0.001	0.70
Emotional wellbeing	N/A	N/A	55.56 (20.07)	53.72, 57.55	N/A	N/A	71.94 (17.47)	70.33, 73.51	13.71 (966.83)	<0.001	0.87
Social functioning	N/A	N/A	61.30 (28.26)	58.83, 63.94	N/A	N/A	83.28 (21.66)	81.41, 84.99	13.74 (918.22)	<0.001	0.87
Pain	N/A	N/A	70.42 (24.52)	68.21, 72.58	N/A	N/A	77.77 (20.17)	76.03, 79.52	5.16 (947.61)	<0.001	0.33
General health	N/A	N/A	59.93 (22.08)	57.77, 62.15	N/A	N/A	66.39 (20.12)	64.64, 68.18	4.82 (978.57)	<0.001	0.31

**TABLE 6:** Descriptive statistics and between-group tests of difference for misophonic responses, psychosocial variables, and quality of life at follow-up.

Measure	Misophonia (*n* = 127)				General population (*n* = 95)				Tests of difference between groups at follow-up		
	Item mean (*SD*)	Lower CI, upper CI	Total mean (*SD*)	Lower CI, upper CI	Item mean (*SD*)	Lower CI, upper CI	Total mean (*SD*)	Lower CI, upper CI	*t* (df)	*p*	Cohen’s *d*
**MRS stand-alone items**											
Frequency	3.90 (1.37)	3.66, 4.13	N/A	N/A	1.62 (0.87)	1.48, 1.78	N/A	N/A	15.10 (214.30)	<0.001	1.92
Recovery	3.89 (1.31)	3.68, 4.11	N/A	N/A	2.51 (1.40)	2.21, 2.77	N/A	N/A	7.59 (220)	<0.001	1.03
Avoidance	5.54 (1.66)	5.25, 5.82	N/A	N/A	2.25 (1.70)	1.98, 2.56	N/A	N/A	14.42 (220)	<0.001	1.96
**MRS subscales**											
Emotional	5.40 (1.26)	5.18, 5.60	37.79 (8.84)	36.32, 39.26	2.36 (1.26)	2.10, 2.63	16.53 (8.83)	14.91, 18.31	17.74 (220)	<0.001	2.41
Physiological	3.04 (1.47)	2.79, 3.31	21.27 (10.32)	19.56, 23.12	1.62 (0.82)	1.46, 1.77	11.33 (5.72)	10.30, 12.49	9.14 (204.57)	<0.001	1.15
Participation	4.03 (1.44)	3.78, 4.28	20.13 (7.17)	18.95, 21.22	1.67 (1.11)	1.47, 1.88	8.33 (5.56)	7.34, 9.42	13.82 (219.71)	<0.001	1.81
**MRS summed scores**											
Severity score	4.17 (1.11)	3.97, 4.36	79.19 (21.15)	75.31, 83.10	1.90 (0.94)	1.73, 2.08	36.18 (17.91)	32.63, 39.75	15.99 (220)	<0.001	2.17
Weighted score	19.24 (7.91)	17.68, 20.71	N/A	N/A	4.87 (5.03)	4.02, 5.84	N/A	N/A	16.49 (214.90)	<0.001	2.10
**Psychosocial variables**											
Anger	2.35 (0.67)	2.24, 2.47	14.13 (4.02)	13.45, 14.79	1.82 (0.54)	1.71, 1.94	10.89 (3.21)	10.24, 11.62	6.65 (219.04)	<0.001	0.87
Disgust	3.06 (0.70)	2.94, 3.19	18.37 (4.08)	17.63, 19.14	2.43 (0.54)	2.31, 2.54	14.57 (3.24)	13.89, 15.25	7.49 (220)	<0.001	1.02
Anxiety	2.26 (0.69)	2.14, 2.39	13.56 (4.12)	12.81, 14.32	1.80 (0.70)	1.67, 1.94	10.82 (4.21)	10.01, 11.63	4.85 (220)	<0.001	0.66
Self-esteem	2.87 (0.67)	2.75, 3.00	28.71 (6.71)	27.45, 29.95	3.24 (0.54)	3.13, 3.35	32.36 (5.41)	31.34, 33.54	4.35 (220)	<0.001	0.59
Depression	2.24 (0.62)	2.13, 2.36	22.41 (6.24)	21.27, 23.59	1.75 (0.56)	1.64, 1.85	17.51 (5.56)	16.42, 18.55	6.06 (220)	<0.001	0.82
**SF-36**											
Physical functioning	N/A	N/A	84.88 (19.38)	81.26, 88.56	N/A	N/A	89.58 (14.54)	86.40, 92.63	2.06 (220.00)	0.040	0.27
Role limitations – physical health	N/A	N/A	76.18 (35.89)	69.98, 82.11	N/A	N/A	85.00 (30.16)	78.91, 90.80	1.99 (216.99)	0.048	0.26
Role limitations – emotional problems	N/A	N/A	52.49 (42.11)	45.32, 59.51	N/A	N/A	72.98 (35.83)	65.73, 80.21	3.91 (216.31)	<0.001	0.52
Energy/Fatigue	N/A	N/A	42.01 (21.02)	37.94, 45.69	N/A	N/A	54.79 (21.79)	50.28, 58.92	4.41 (220)	<0.001	0.60
Emotional wellbeing	N/A	N/A	57.42 (20.07)	53.65, 61.30	N/A	N/A	72.25 (17.30)	68.95, 75.47	5.78 (220)	<0.001	0.78
Social functioning	N/A	N/A	67.72 (28.19)	62.73, 72.48	N/A	N/A	84.61 (18.64)	80.31, 88.25	5.36 (216.96)	<0.001	0.69
Pain	N/A	N/A	72.52 (22.97)	68.49, 76.31	N/A	N/A	77.58 (18.69)	73.48, 81.13	1.81 (218.42)	0.072	0.24
General health	N/A	N/A	63.66 (19.86)	59.97, 67.29	N/A	N/A	68.05 (22.31)	63.55, 72.69	1.55 (220)	0.12	0.21

**TABLE 7:** Within-participant tests of difference over time for misophonic responses, psychosocial variables, and quality of life.

Measure	Misophonia							General population						
	Baseline (*n* = 127)		Follow-up (*n* = 127)		Tests of within-participant difference over time			Baseline (*n* = 95)		Follow-up (*n* = 95)		Tests of within-participant difference over time		
	Item mean (*SD*)	Lower CI, upper CI	Item mean (*SD*)	Lower CI, upper CI	*t*(df)	*p*	Cohen’s *d*	Item mean (*SD*)	Lower CI, upper CI	Item mean (*SD*)	Lower CI, upper CI	*t* (df)	*p*	Cohen’s *d*
**MRS stand-alone items**														
Frequency	4.12 (1.19)	3.92, 4.31	3.90 (1.37)	3.66, 4.13	2.82 (126)	0.006	0.25	1.48 (0.80)	1.34, 1.64	1.62 (0.87)	1.48, 1.78	1.53 (94)	0.13	0.16
Recovery	4.00 (1.32)	3.76, 4.26	3.89 (1.31)	3.68, 4.11	1.19 (126)	0.24	0.11	2.27 (1.43)	2.00, 2.54	2.51 (1.40)	2.21, 2.77	1.51 (94)	0.13	0.16
Avoidance	5.65 (1.59)	5.37, 5.91	5.54 (1.66)	5.25, 5.82	0.96 (126)	0.34	0.09	2.22 (1.82)	1.87, 2.53	2.25 (1.70)	1.98, 2.56	0.16 (94)	0.88	0.02
**MRS subscales**														
Emotional	5.72 (1.01)	5.54, 5.90	5.40 (1.26)	5.18, 5.60	3.95 (126)	<0.001	0.35	2.21 (1.24)	1.98, 2.44	2.36 (1.26)	2.10, 2.63	1.29 (94)	0.20	0.13
Physiological	3.19 (1.37)	2.96, 3.41	3.04 (1.47)	2.79, 3.31	1.83 (126)	0.070	0.16	1.54 (0.81)	1.39, 1.68	1.62 (0.82)	1.46, 1.77	1.08 (94)	0.28	0.11
Participation	4.15 (1.37)	3.91, 4.40	4.03 (1.44)	3.78, 4.28	1.30 (126)	0.20	0.12	1.56 (0.93)	1.39, 1.73	1.67 (1.11)	1.47, 1.88	0.97 (94)	0.34	0.10
**MRS summed scores**														
Severity score	4.37 (0.98)	4.19, 4.54	4.17 (1.11)	3.97, 4.36	3.37 (126)	0.001	0.30	1.79 (0.91)	1.62, 1.98	1.90 (0.94)	1.73, 2.08	1.32 (94)	0.19	0.14
Weighted score	20.72 (7.65)	19.25, 22.17	19.24 (7.91)	17.68, 20.71	3.47 (126)	0.001	0.31	4.41 (4.80)	3.47, 5.30	4.87 (5.03)	4.02, 5.84	0.97 (94)	0.33	0.10
**Psychosocial variables**														
Anger	2.38 (0.67)	2.26, 2.50	2.35 (0.67)	2.24, 2.47	0.58 (126)	0.56	0.05	1.73 (0.52)	1.63, 1.84	1.82 (0.54)	1.71, 1.94	1.97 (94)	0.05	0.20
Disgust	3.14 (0.69)	3.02, 3.27	3.06 (0.70)	2.94, 3.19	1.61 (126)	0.11	0.14	2.42 (0.54)	2.30, 2.54	2.43 (0.54)	2.31, 2.54	0.16 (94)	0.88	0.02
Anxiety	2.36 (0.68)	2.24, 2.47	2.26 (0.69)	2.14, 2.39	1.75 (126)	0.082	0.16	1.63 (0.60)	1.52, 1.74	1.80 (0.70)	1.67, 1.94	2.65 (94)	0.009	0.27
Self-esteem	2.78 (0.65)	2.66, 2.91	2.87 (0.67)	2.75, 3.00	2.53 (126)	0.013	0.22	3.30 (0.52)	3.20, 3.40	3.24 (0.54)	3.13, 3.35	2.03 (94)	0.045	0.21
Depression	2.32 (0.58)	2.22, 2.42	2.24 (0.62)	2.13, 2.36	1.82 (126)	0.071	0.16	1.68 (0.51)	1.57, 1.79	1.75 (0.56)	1.64, 1.85	1.97 (94)	0.052	0.20
SF-36*														
Physical functioning	85.47 (19.93)	81.56, 88.79	84.88 (19.38)	81.26, 88.56	0.69 (126)	0.49	0.06	90.68 (12.15)	87.85, 93.05	89.58 (14.54)	86.40, 92.63	1.32 (94)	0.19	0.14
Role limitations – physical health	72.83 (39.34)	66.27, 78.88	76.18 (35.89)	69.98, 82.11	1.00 (126)	0.32	0.09	91.32 (24.67)	85.67, 95.88	85.00 (30.16)	78.91, 90.80	2.34 (94)	0.019	0.25
Role limitations – emotional problems	48.82 (43.20)	41.01, 55.71	52.49 (42.11)	45.32, 59.51	1.02 (126)	0.31	0.09	78.60 (35.37)	71.37, 85.54	72.98 (35.83)	65.73, 80.21	1.39 (94)	0.17	0.14
Energy/Fatigue	40.98 (20.87)	37.19, 44.62	42.01 (21.02)	37.94, 45.69	0.65 (126)	0.52	0.06	58.74 (20.63)	54.58, 62.32	54.79 (21.79)	50.28, 58.92	2.48 (94)	0.015	0.25
Emotional wellbeing	55.40 (19.85)	52.03, 58.86	57.42 (20.07)	53.65, 61.30	1.54 (126)	0.13	0.14	76.08 (16.59)	72.51, 80.04	72.25 (17.30)	68.95, 75.47	2.89 (94)	0.005	0.30
Social functioning	62.99 (27.57)	58.17, 67.74	67.72 (28.19)	62.73, 72.48	2.28 (126)	0.024	0.20	88.42 (19.57)	83.63, 92.42	84.61 (18.64)	80.31, 88.25	2.01 (94)	0.047	0.21
Pain	71.56 (22.95)	67.59, 75.51	72.52 (22.97)	68.49, 76.31	0.58 (126)	0.56	0.05	80.16 (17.44)	76.41, 83.97	77.58 (18.69)	73.48, 81.13	1.68 (94)	0.097	0.17
General health	62.20 (20.78)	58.53, 65.94	63.66 (19.86)	59.97, 67.29	1.20 (126)	0.23	0.11	68.11 (19.71)	64.09, 72.00	68.05 (22.31)	63.55, 72.69	0.04 (94)	0.97	0.004

*Total means reported for SF36 subscales.

**TABLE 8:** Correlation coefficients between misophonic responses, psychosocial variables, and quality of life for misophonia group at baseline.

	MRS stand-alone items			MRS subscales			MRS summed scores			Psychosocial variables				SF-36							
	1	2	3	4	5	6	7	8	9	10	11	12	13	14	15	16	17	18	19	20	21
1. Frequency	–	–	–	–	–	–	–	–	–	–	–	–	–	–	–	–	–	–	–	–	–
2. Recovery	0.19***	–	–	–	–	–	–	–	–	–	–	–	–	–	–	–	–	–	–	–	–
3. Avoidance	0.24***	0.22***	–	–	–	–	–	–	–	–	–	–	–	–	–	–	–	–	–	–	–
4. Emotional	0.36***	0.28***	0.43***	–	–	–	–	–	–	–	–	–	–	–	–	–	–	–	–	–	–
5. Physiological	0.30***	0.47***	0.29***	0.46***	–	–	–	–	–	–	–	–	–	–	–	–	–	–	–	–	–
6. Participation	0.50***	0.37***	0.48***	0.49***	0.50***	–	–	–	–	–	–	–	–	–	–	–	–	–	–	–	–
7. Severity score	0.46***	0.47***	0.48***	0.79***	0.84***	0.79***	–	–	–	–	–	–	–	–	–	–	–	–	–	–	–
8. Weighted score	0.61***	0.61***	0.64***	0.69***	0.76***	0.78***	0.92***	–	–	–	–	–	–	–	–	–	–	–	–	–	–
9. Anger	0.25***	0.33***	0.19***	0.31***	0.39***	0.40***	0.45***	0.46***	–	–	–	–	–	–	–	–	–	–	–	–	–
10. Disgust	0.39***	0.26***	0.18***	0.41***	0.33***	0.36***	0.45***	0.46***	0.40***	–	–	–	–	–	–	–	–	–	–	–	–
11. Anxiety	0.24***	0.30***	0.09	0.24***	0.32***	0.33***	0.39***	0.39***	0.45***	0.28***	–	–	–	–	–	–	–	–	–	–	–
12. Self-esteem	–0.25***	–0.25***	–0.07	–0.23***	–0.26***	–0.32***	–0.33***	–0.33***	–0.42***	–0.26***	–0.61***	–	–	–	–	–	–	–	–	–	–
13. Depression	0.31***	0.31***	0.20***	0.29***	0.36***	0.42***	0.44***	0.46***	0.46***	0.36***	0.70***	–0.68***	–	–	–	–	–	–	–	–	–
14. Physical functioning	–0.19***	–0.13**	–0.17***	–0.08	–0.21***	–0.20***	–0.20***	–0.25***	–0.17***	–0.15**	–0.22***	0.22***	–0.31***	–	–	–	–	–	–	–	–
15. Role limitations – physical health	–0.15**	–0.20***	–0.14**	–0.03	–0.17***	–0.18***	–0.16***	–0.23***	–0.22***	–0.12**	–0.27***	0.25***	–0.40***	0.53***	–	–	–	–	–	–	–
16. Role limitations –emotional problems	–0.21***	–0.23***	–0.10*	–0.23***	–0.26***	–0.29***	–0.32***	–0.32***	–0.37***	–0.21***	–0.43***	0.47***	–0.59***	0.15***	0.34***	–	–	–	–	–	–
17. Energy/Fatigue	–0.25***	–0.26***	0-.10*	–0.23***	–0.30***	–0.30***	–0.34***	–0.35***	–0.32***	–0.21***	–0.54***	0.57***	0.70***	0.36***	0.44***	0.53***	–	–	–	–	–
18. Emotional wellbeing	–0.28***	–0.30***	–0.16***	–0.30***	–0.33***	–0.38***	–0.42***	–0.43***	–0.48***	–0.29***	–0.73***	0.69***	–0.81***	0.25***	0.35***	0.60***	0.70***	–	–	–	–
19. Social functioning	–0.36***	–0.35***	–0.22***	–0.29***	–0.40***	–0.47***	–0.48***	–0.51***	–0.44***	–0.30***	–0.51***	0.48***	–0.64***	0.40***	0.46***	0.56***	0.56***	0.63***	–	–	–
20. Pain	–0.18***	–0.23***	–0.14**	–0.05	–0.24***	–0.22***	–0.21***	–0.27***	–0.18***	–0.19***	–0.29***	0.27***	–0.40***	0.62***	0.61***	0.24***	0.44***	0.33***	0.44***	–	–
21. General health	–0.21***	–0.25***	–0.08	–0.08	–0.25***	–0.26***	–0.24***	–0.28***	–0.31***	–0.22***	–0.40***	0.43***	–0.48***	0.55***	0.55***	0.33***	0.56***	0.47***	0.47***	0.56***	–

*p is significant < 0.05; **p is significant < 0.01;*** p is significant < 0.001.

#### Differences between groups at baseline

At baseline, compared to those in the general population, people with self-reported misophonia experienced triggers more frequently, took longer to recover from triggers, and reported avoiding situations/environments to a greater extent in order to avoid triggers (all *p* < 0.001; Table 5). Those with misophonia also reported greater emotional and physiological responses to triggers than those in the general population, as well as a greater impact of triggers on their participation in everyday life (all *p* < 0.001; Table 5). The differences between these two groups on MRS scores were all represented by large effect sizes (*d* range = 1.18–2.95; Table 5). Even though misophonia is a condition which is yet to be formally recognized, these results show a clear difference between those who experience an extreme response to a trigger and those who do not.

#### Differences between groups at follow-up

Statistically significant differences were also seen in MRS scores at follow-up between the misophonia and general population groups; these differences were again represented by large effect sizes (all *p* < 0.001; Table 6). As at baseline, people with self-reported misophonia experienced triggers more frequently, took longer to recover from triggers, and reported avoiding situations/environments to a greater extent in order to avoid triggers. They also reported greater emotional and physiological responses to triggers and a greater impact of triggers on their participation in everyday life.

#### Within-participant differences over time

For four elements of the MRS (recovery, avoidance, physiological responses, participation) there were no statistically significant differences over time amongst the misophonia group (Table 7). There were, however, statistically significant differences on two elements of the MRS for those with self-reported misophonia; at follow-up, people with misophonia reported reduced frequency of triggers (*p* = 0.006) and reductions in the extent to which they experienced emotional responses (*p* < 0.001). There were also significant differences over time for severity and weighted scores of the MRS in those with misophonia (*p* = 0.001); these composite scores improved, presumably as a result of the reductions in frequency of triggers and the strength of emotional responses. There were no statistically significant differences over time amongst the general population group on the MRS (Table 7).

### Psychosocial variables: Differences between and within participants

Descriptive statistics for anger, disgust, anxiety, self-esteem, and depression at baseline and follow-up for those with and without self-reported misophonia are reported in Tables 5–7.

#### Differences between groups at baseline

At baseline, levels of anger, disgust, anxiety, and depression were all higher in those with self-reported misophonia, while self-esteem was lower in those with misophonia; these differences were all represented by large effect sizes (all *p* < 0.001; Table 5).

#### Differences between groups at follow-up

The same pattern as at baseline is present at follow-up, with levels of anger, disgust, anxiety, and depression all higher in those with self-reported misophonia, and self-esteem lower in those with misophonia (all *p* < 0.001; Table 6). At follow-up, differences in self-esteem and anxiety between those with and without misophonia were represented by medium effect sizes, while differences in anger, disgust, and depression were represented by large effect sizes (Table 6).

#### Within-participant differences over time

Longitudinally, there were no differences in anger, disgust, anxiety or depression in the misophonia group (Table 7). There was, however, a statistically significant increase in self-esteem at follow-up compared to baseline in those with self-reported misophonia [*t*(126) = 2.53, *p* = 0.013; *d* = 0.22]. In the general population group, there were no statistically significant differences in anger, disgust, anxiety, or depression over time. There was, however, a statistically significant decrease in self-esteem over time amongst those in the general population [*t*(94) = 2.65, *p* = 0.009; *d* = 0.27].

### Quality of life: Differences between and within participants

Descriptive statistics for quality of life at baseline and follow-up for those with and without self-reported misophonia are reported in Tables 5–7.

#### Differences between groups at baseline

At baseline, quality of life was lower in those with self-reported misophonia compared to the general population across all eight SF-36 subscales (physical functioning *p* = 0.004, all other *p* < 0.001; Table 5). The effect sizes for these differences were very small for physical functioning, small for physical health role limitations, pain, and general health, medium for role limitations due to emotional problems and energy/fatigue, and large for emotional wellbeing and social functioning. This shows that quality of life for people with misophonia is worse than for those without misophonia especially for the role limitations due to emotional problems, emotional well-being, energy/fatigue, and social functioning, which highlights the emotional impact of the condition and the impact on social interactions.

#### Differences between groups at follow-up

At follow-up, quality of life was again lower in those with self-reported misophonia compared to the general population across all subscales, but these differences were only statistically significant for four subscales; the data show greater differences in role limitations due to emotional problems, energy/fatigue, emotional wellbeing, and social functioning, with differences represented by medium effect sizes (Table 6). In line with the baseline data, quality of life at follow-up was lower for those with misophonia than those without.

#### Within-participant differences over time

Examining the longitudinal data for those in the misophonia group, there were no statistically significant differences in quality of life on any of the eight SF-36 sub-scales between baseline and follow-up. For those in the general population group, there were statistically significant decreases in quality of life on two SF-36 sub-scales: energy/fatigue [*t*(94) = 2.48, *p* = 0.015, *d* = 0.25)] and emotional wellbeing [*t*(94) = 2.89, *p* = 0.005, *d* = 0.30)]. For the other six sub-scales there were no statistically significant differences in quality of life for those in the general population (Table 7).

#### Comparing quality of life in misophonia to other conditions

To further examine how living with misophonia compares to living with other conditions, the SF-36 scores for people with self-reported misophonia in the current study are shown in Table 9, alongside the SF-36 scores of people in the general population (current study), people with long-term conditions (Bowling et al., 1999), obsessive compulsive disorder (OCD) (Rodriguez-Salgado et al., 2006) and tinnitus (Ross et al., 2007). People with misophonia score low on role limitations due to emotional problems, fatigue/vitality, emotional wellbeing, and social functioning in comparison to general population samples. People with misophonia score higher than people with OCD, but lower than people with tinnitus and other long-term conditions, on role limitations due to emotional problems, energy/fatigue and social functioning.

**TABLE 9:** Quality of life total means (and standard deviations) of people with misophonia, general population, tinnitus, obsessive compulsive disorder (OCD) and long-term conditions.

SF-36 subscale	Misophonia (current study, baseline)	General population (current study, baseline)	Obsessive compulsive disorder (Rodriguez-Salgado et al., 2006)	General population (Rodriguez-Salgado et al., 2006)	Tinnitus (Ross et al., 2007)	Long-term conditions (Bowling et al., 1999)*	General population (Bowling et al., 1999)*
Physical functioning	84.77 (20.46)	88.15 (15.96)	80.1 (22.7)	84.7 (24.0)	81.58 (19.68)	52.3 (28.9)	92.7 (20.2)
Role limitations – physical health	73.57 (37.90)	85.24 (29.43)	54.3 (41.4)	83.2 (35.2)	56.13 (4.09)	44.1 (42.5)	90.9 (24.7)
Role limitations – emotional problems	**49.90 (43.40)**	74.29 (37.07)	35.4 (44.4)	88.6 (30.1)	58.34 (43.52)	71.6 (41.7)	92.1 (23.5)
Energy/Fatigue	**41.47 (21.56)**	56.47 (21.31)	40.6 (19.6)	66.9 (22.1)	42.88 (18.92)	44.1 (23.9)	69.0 (17.9)
Emotional wellbeing	**55.56 (20.07)**	71.94 (17.47)	45.7 (19.9)	73.3 (20.1)	54.01 (17.55)	67.5 (22.1)	79.8 (16.1)
Social functioning	**61.30 (28.26)**	83.28 (21.66)	48.8 (32.6)	90.1 (20.0)	67.10 (22.92)	65.3 (30.5)	93.5 (14.9)
Pain	70.42 (24.52)	77.77 (20.17)	75.6 (26.2)	79.0 (27.9)	61.85 (28.46)	56.0 (30.2)	87.4 (19.6)
General health	59.93 (22.08)	66.39 (20.12)	51.0 (22.2)	68.3 (22.3)	52.87 (17.15)	44.8 (23.5)	79.0 (16.1)

*Normative data for the SF-36.

### Predicting misophonic response at baseline and follow-up – misophonia group only

The regression analyses which examined the underlying emotions associated with misophonia (cross-sectionally and longitudinally) are presented in Table 10. Age was associated with some aspects of the misophonic response; those of a younger age experienced a stronger emotional and physiological response. Although depression and anxiety were significant predictors of some aspects of misophonic responses cross-sectionally (i.e., at baseline, with effects of anxiety generally disappearing after depression was added to the models), when predicting misophonic responses longitudinally, both depression and anxiety did not emerge as significant predictors of any of the misophonic response variables. In other words, depression and anxiety do not predict experiences of misophonia over time.

**TABLE 10:** Summary of hierarchical regression models for predicting misophonic response cross-sectionally (at baseline) and longitudinally (at follow-up) in misophonia group.

	Frequency			Recovery			Avoidance			Emotional response			Physiological response			Participation			Severity score			Weighted score		
	*B*	*p*	Δ *R*^2^	*B*	*p*	Δ *R*^2^	*B*	*p*	Δ *R*^2^	*B*	*p*	Δ *R*^2^	*B*	*p*	Δ *R*^2^	*B*	*p*	Δ *R*^2^	*B*	*p*	Δ *R*^2^	*B*	*p*	Δ *R*^2^
**Baseline**																								
**Block 1**																								
Age	−0.21	<0.001		−0.12	0.006		<-0.01	0.98		−0.23	<0.001		0.20	<0.001		−0.11	0.017		−0.23	<0.001		−0.21	<0.001	
**Block 2**			0.15***			0.14***			0.05***			0.16***			0.18***			0.22***			0.27***			0.29***
Age	−0.14	0.001		−0.05	0.27		0.05	0.29		−0.14	0.001		−0.11	0.006		−0.01	0.86		−0.11	0.003		−0.09	0.018	
Anxiety	0.12	0.012		0.17	<0.001		−0.01	0.81		0.08	0.086		0.16	<0.001		0.16	<0.001		0.17	<0.001		0.16	<0.001	
Anger	0.05	0.33		0.19	<0.001		0.15	0.004		0.12	0.010		0.23	<0.001		0.24	<0.001		0.24	<0.001		0.25	<0.001	
Disgust	0.31	<0.001		0.12	0.008		0.13	0.006		0.31	<0.001		0.18	<0.001		0.22	<0.001		0.29	<0.001		0.30	<0.001	
**Block 3**			0.01**			**0.005**			0.02**			**0.004**			0.01*			0.03***			0.02***			0.03***
Age	−0.13	0.002		−0.05	0.28		0.05	0.25		−0.14	0.001		−0.11	0.007		<−0.01	0.94		−0.11	0.004		−0.09	0.022	
Anxiety	0.02	0.72		0.11	0.059		−0.13	0.034		0.02	0.72		0.08	0.19		0.02	0.77		0.05	0.34		0.02	0.73	
Anger	0.03	0.59		0.18	<0.001		0.13	0.017		0.11	0.023		0.21	<0.001		0.21	<0.001		0.22	<0.001		0.22	<0.001	
Disgust	0.29	<0.001		0.11	0.021		0.11	0.031		0.30	<0.001		0.16	<0.001		0.19	<0.001		0.26	<0.001		0.27	<0.001	
Depression	0.16	0.008		0.10	0.098		0.20	0.002		0.10	0.11		0.14	0.016		0.24	<0.001		0.19	<0.001		0.24	<0.001	
**Follow-up**																								
**Block 1**																								
Age	−0.15	0.084		−0.08	0.37		0.01	0.88		−0.23	0.011		−0.33	<0.001		−0.19	0.034		−0.31	<0.001		−0.25	0.004	
**Block 2**			0.10**			0.12**			0.06* ^†^			0.10**			0.10**			0.17***			0.17***			0.20***
Age	−0.11	0.21		−0.05	0.56		0.02	0.81		−0.21	0.016		−0.30	<0.001		−0.14	0.092		−0.28	0.001		−0.21	0.008	
Anxiety	0.15	0.10		0.004	0.97		−0.11	0.25		−0.08	0.38		0.031	0.73		0.09	0.29		0.01	0.87		0.02	0.84	
Anger	0.12	0.23		0.29	0.003		0.20	0.048		0.19	0.054		0.23	0.013		0.19	0.045		0.25	0.005		0.32	0.001	
Disgust	0.18	0.056		0.11	0.23		0.12	0.19		0.21	0.019		0.15	0.091		0.27	0.002		0.25	0.003		0.22	0.010	
**Block 3**			**0.01**			**0.02**			**0.003** ^†^			**<0.01**			**0.01**			**0.004**			**0.006**			**0.01**
Age	−0.10	0.26		−0.04	0.68		0.02	0.87		−0.21	0.017		−0.28	0.001		−0.13	0.11		−0.27	0.001		−0.20	0.013	
Anxiety	0.08	0.53		−0.11	0.36		−0.06	0.63		−0.08	0.51		−0.07	0.54		0.04	0.74		−0.05	0.63		−0.06	0.56	
Anger	0.09	0.35		0.26	0.011		0.21	0.039		0.19	0.060		0.20	0.035		0.17	0.076		0.23	0.011		0.29	0.002	
Disgust	0.15	0.099		0.08	0.40		0.14	0.15		0.21	0.022		0.12	0.18		0.25	0.005		0.23	0.007		0.20	0.022	
Depression	0.13	0.30		0.19	0.13		−0.08	0.52		−0.01	0.97		0.17	0.16		0.09	0.44		0.11	0.34		0.14	0.24	

F-ratios at block 2 range from 6.77 to 60.33 at baseline and 2.07 to 11.41 at follow-up; F-ratios at block 3 range from 7.47 to 54.05 at baseline and 1.73 to 12.27 at follow-up.

*p is significant < 0.05; **p is significant < 0.01; ***p is significant < 0.001.

^†^Overall model not significant at p < 0.0167.

Anger and disgust did, however, emerge as significant predictors of several aspects of misophonic response, both cross-sectionally and longitudinally, even after controlling for depression. Higher levels of anger at baseline predicted taking a longer time to recover from triggers and a stronger physiological response at follow-up. Stronger feelings of disgust at baseline predicted a stronger emotional response to the trigger and more perceived impact on participation in life at follow-up. Stronger feelings of anger and disgust at baseline were both predictive of the summary MRS scores (both severity and weighted scores) at follow-up. No significant variables emerged for predicting frequency of triggers at follow-up and while anger did emerge as a significant predictor for avoidance at follow-up, the overall model was not significant.

### Predicting quality of life at baseline and follow-up – misophonia group only

The regression analyses, which examine the association of misophonic responses with quality of life (cross-sectionally and longitudinally), are presented in Table 11. Cross-sectionally, some aspects of the misophonic response were predictive of all eight quality of life domains, but once the psychosocial variables were included in the model some of these effects disappeared, and misophonia was only predictive across five domains: physical functioning, role limitations due to physical health, social functioning, pain, and general health. Lower scores on the Physical Functioning domain at baseline, showing worse physical functioning, was predicted by older age, more frequent misophonic responses, more avoidance of triggers, a stronger physiological response to the trigger, and more depressive symptoms. Greater role limitations due to physical health at baseline were predicted by older age, longer recovery after a misophonic response, a reduced emotional response to a trigger, and more depressive symptoms. Worse social functioning at baseline was predicted by more frequent responses to triggers, a greater physiological response to the trigger, greater perceived impact on participation in life, and more depressive symptoms. Higher pain levels at baseline were predicted by a higher frequency of response to triggers, a reduced emotional response, an increased physiological response, and more depressive symptoms. Reduced general health at baseline was predicted by a reduced emotional response to triggers, higher self-esteem, and greater depressive symptoms. While all the MRS misophonia items and subscales were predictive of quality of life cross-sectionally, the frequency of the trigger, the emotional response and the physiological response were predictive of three of the quality of life domains, suggesting a wider impact of these aspects of misophonia.

**TABLE 11:** Summary of hierarchical regression models for predicting quality of life cross-sectionally (at baseline) and longitudinally (at follow-up) in misophonia group.

	Physical functioning			Role limitations – physical health			Role limitations −emotional problems			Energy/Fatigue			Emotional wellbeing			Social functioning			Pain			General health		
	*B*	*p*	Δ *R*^2^	*B*	*p*	Δ *R*^2^	*B*	*p*	Δ *R*^2^	*B*	*p*	Δ *R*^2^	*B*	*p*	Δ *R*^2^	*B*	*p*	Δ *R*^2^	*B*	*p*	Δ *R*^2^	*B*	*p*	Δ *R*^2^
**Baseline**																								
**Block 1**																								
Age	–0.07	0.14		–0.08	0.066		0.21	<0.001		0.15	0.001		0.17	<0.001		0.15	0.001		–0.07	0.12		0.04	0.41	
**Block 2**			0.09***			0.09***			0.10***			0.13***			0.18***			0.27***			0.12***			0.12***
Age	–0.12	0.01		–0.12	0.008		0.13	0.003		0.06	0.16		0.07	0.090		0.04	0.27		–0.13	0.006		–0.02	0.59	
Frequency	–0.15	0.005		–0.11	0.027		–0.05	0.33		–0.10	0.052		–0.08	0.097		–0.14	0.002		–0.13	0.008		–0.12	0.018	
Recovery	–0.03	0.54		–0.14	0.005		–0.11	0.031		–0.12	0.012		–0.14	0.003		–0.14	0.001		–0.13	0.007		–0.14	0.004	
Avoidance	–0.10	0.048		–0.08	0.14		0.07	0.19		0.08	0.10		0.07	0.15		0.02	0.74		–0.05	0.29		0.04	0.41	
Emotional	0.10	0.084		0.14	0.014		–0.07	0.23		–0.04	0.41		–0.09	0.079		0.02	0.71		0.16	0.004		0.13	0.021	
Physiological	–0.16	0.003		–0.11	0.049		–0.07	0.20		–0.13	0.018		–0.10	0.059		–0.15	0.002		–0.18	0.001		–0.15	0.007	
Participation	–0.05	0.45		–0.06	0.31		–0.18	0.003		–0.16	0.009		–0.23	<0.001		–0.29	<0.001		–0.08	0.21		–0.15	0.011	
**Block 3**			0.05***			0.12***			0.24***			0.36***			0.51***			0.19***			0.10***			0.16***
Age	–0.13	0.004		–0.14	0.002		0.11	0.007		0.03	0.45		0.03	0.26		0.02	0.51		–0.14	0.001		–0.06	0.18	
Frequency	–0.12	0.022		–0.07	0.14		0.01	0.76		–0.02	0.59		0.009	0.76		–0.08	0.031		–0.10	0.048		–0.07	0.13	
Recovery	0.004	0.94		–0.09	0.048		–0.03	0.45		–0.03	0.48		–0.01	0.67		–0.08	0.051		–0.09	0.063		–0.08	0.092	
Avoidance	–0.11	0.027		–0.08	0.088		0.04	0.35		0.04	0.26		<0.01	0.97		–0.01	0.80		–0.06	0.19		0.01	0.92	
Emotional	0.11	0.051		0.15	0.005		–0.04	0.34		–0.02	0.69		–0.05	0.091		0.04	0.36		0.17	0.001		0.15	0.003	
Physiological	–0.13	0.017		–0.06	0.27		<0.01	0.99		–0.04	0.32		0.01	0.74		–0.08	0.048		0.14	0.009		–0.09	0.061	
Participation	0.02	0.74		0.03	0.60		–0.04	0.46		0.02	0.69		–0.02	0.66		–0.16	0.001		0.01	0.83		–0.04	0.52	
Self–esteem	0.05	0.45		–0.02	0.79		0.10	0.051		0.14	0.002		0.19	<0.001		–0.04	0.37		0.02	0.78		0.18	0.002	
Anxiety	0.01	0.82		–0.03	0.67		0.01	0.86		–0.05	0.30		–0.27	<0.001		–0.07	0.16		0.01	0.88		–0.07	0.20	
Depression	–0.24	<0.001		–0.42	<0.001		–0.49	<0.001		–0.55	<0.001		–0.47	<0.001		–0.43	<0.001		–0.35	<0.001		–0.26	<0.001	
**Follow-up**																								
**Block 1**																								
Age	–0.14	0.13		–0.04	0.64		0.28	0.001		0.26	0.003		0.26	0.003		0.13	0.16		–0.05	0.56		0.05	0.60	
**Block 2**			0.10* ^†^			**0.07** ^†^			0.12**			0.11*			0.12*			0.11* ^†^			0.12* ^†^			**0.10** ^†^
Age	–0.21	0.021		–0.09	0.36		0.18	0.039		0.19	0.036		0.17	0.051		0.05	0.58		–0.14	0.12		–0.02	0.85	
Frequency	–0.20	0.05		–0.10	0.33		0.09	0.34		–0.07	0.47		–0.15	0.13		–0.08	0.40		–0.02	0.81		–0.17	0.087	
Recovery	–0.14	0.17		–0.19	0.074		–0.05	0.58		–0.15	0.13		–0.15	0.12		–0.16	0.11		–0.06	0.57		–0.15	0.15	
Avoidance	0.05	0.64		0.08	0.49		0.34	0.002		0.11	0.34		0.14	0.22		0.02	0.85		0.21	0.067		0.23	0.048	
Emotional	0.04	0.70		0.05	0.64		–0.30	0.005		–0.22	0.044		–0.11	0.31		–0.03	0.79		0.09	0.42		0.04	0.73	
Physiological	–0.13	0.23		0.04	0.75		0.05	0.65		0.12	0.26		0.02	0.83		–0.09	0.42		–0.19	0.083		0.08	0.50	
Participation	–0.04	0.74		–0.13	0.30		–0.29	0.015		–0.17	0.15		–0.18	0.14		–0.13	0.31		–0.29	0.019		–0.18	0.15	
**Block 3**			**0.05**			0.09** ^†^			0.21***			0.15***			0.31***			0.10**			**0.05**			0.07* ^†^
Age	–0.23	0.012		–0.12	0.20		0.14	0.071		0.16	0.052		0.15	0.039		0.03	0.76		–0.17	0.071		–0.04	0.70	
Frequency	–0.16	0.11		–0.05	0.62		0.16	0.053		–0.01	0.93		–0.06	0.41		–0.03	0.75		0.01	0.91		–0.13	0.18	
Recovery	–0.07	0.48		–0.12	0.27		0.08	0.38		–0.03	0.73		0.01	0.91		–0.07	0.46		<0.01	1.00		–0.07	0.50	
Avoidance	0.01	0.94		0.06	0.59		0.28	0.006		0.02	0.85		–0.02	0.79		–0.04	0.71		0.19	0.11		0.18	0.14	
Emotional	0.06	0.58		0.03	0.78		–0.30	0.003		–0.17	0.097		<0.01	0.99		< –0.01	0.97		0.09	0.45		0.07	0.55	
Physiological	–0.10	0.35		0.10	0.38		0.12	0.21		0.17	0.093		0.07	0.40		–0.04	0.72		–0.15	0.17		0.11	0.34	
Participation	<–0.01	0.97		–0.11	0.36		–0.23	0.031		–0.10	0.36		–0.06	0.55		–0.08	0.52		–0.27	0.031		–0.13	0.29	
Self–esteem	–0.01	0.96		–0.21	0.12		–0.13	0.25		0.02	0.85		0.18	0.077		–0.05	0.71		–0.10	0.44		0.02	0.89	
Anxiety	–0.04	0.72		–0.12	0.35		0.01	0.94		–0.07	0.53		–0.19	0.042		–0.15	0.21		–0.06	0.63		–0.04	0.78	
Depression	–0.22	0.12		–0.37	0.011		–0.60	<0.001		–0.38	0.004		–0.34	0.002		–0.29	0.040		–0.26	0.070		–0.26	0.076	

F-ratios at block 2 range from 6.98 to 28.77 at baseline and 1.24 to 4.33 at follow-up; F-ratios at block 3 range from 8.21 to 125.75 at baseline and 2.22 to 11.54 at follow-up.

*p is significant < 0.05; **p is significant < 0.01; ***p is significant < 0.001.

^†^Overall model not significant at p < 0.0167.

The longitudinal regression results provide a clearer understanding of the effects of misophonia on quality of life over time. In the baseline analyses, depression was predictive of quality of life across all eight domains; however, at follow-up depression predicted quality of life for only five domains (all but physical functioning, pain and general health). The misophonic response variables were predictive of quality of life over time but for only two domains (role limitations due to emotional problems and pain). Less avoidance of triggers, a greater emotional response to the trigger, a greater perception of impact on participation in life, and higher depression at baseline were associated with more role limitations due to emotional problems at follow-up. Greater pain at follow-up was predicted by greater perceived impact on participation in life at baseline. Finally, a greater emotional response at baseline was associated with increased fatigue at follow-up; however, this relationship was only evident at block 2, and disappeared in block 3 (with depression again being the significant psychosocial predictor).

## Discussion

This paper presents the results of a large-scale, longitudinal, online survey that examined the role of negative emotions in the experience of misophonia, compared the quality of life in people with and without self-reported misophonia, and examined the impact of misophonia on quality of life over time. Our results expand our understanding of the characteristics of misophonia and of the quality of life for a person with misophonia, and it is clear that people with self-reported misophonia experience different responses to triggers compared to people in the general population.

Regarding the type of triggers that bring about a misophonic response, our study suggests that while triggers are predominantly auditory and visual, other sensory triggers may also be experienced. In our sample, around a quarter of those with self-reported misophonia also reported tactile and olfactory triggers (26.7 and 24.7% respectively), with a smaller proportion also reporting taste and ‘other’ sensory triggers. These results give weight to other studies that have suggested triggers may be of any sensory stimuli and not limited to only auditory or visual stimuli (Dozier, 2015; Dozier et al., 2017; Zhou et al., 2017; Brout et al., 2018; Jager et al., 2020; Swedo et al., 2022). These results broaden our understanding of misophonic triggers, but further research is needed to ensure that being triggered by other sensory stimuli does not, in fact, indicate the presence of a different or comorbid condition.

People with self-reported misophonia perceived their working environment to be noisier than those without misophonia, despite there being no reported difference in work environments in terms of situation (at home) and number of people in the working environment. Little research has been conducted in the work domain in relation to misophonia, but in support of our results a study with undergraduate students showed a significant positive correlation between misophonia scores and impairment at work/school (Wu et al., 2014).

Finally, our sample reported no change in misophonic scores over time, which contradicts findings from other studies where participants have reported perceived changes over time (Bernstein et al., 2013; Edelstein et al., 2013; Kluckow et al., 2014; Rouw and Erfanian, 2018). However, this may be due to differences in measurement as some studies ask participants about their perceptions of change, whereas in this study we did not ask about perceived change, but instead measured the misophonic response at two different time points.

### Negative emotions and misophonia

One of the aims this research was to determine the association of anger, disgust and anxiety with misophonia. In our study, people with self-reported misophonia experienced greater negative emotions than those in the general population with scores on anger, disgust and anxiety significantly higher than in those without misophonia (the general population). In addition, depressive symptoms were higher, and self-esteem was lower, in those with misophonia than in the general population. These differences in psychosocial variables supports research from other conditions where a similar pattern is evident (for example, Brueggemann et al., 2022).

Previous literature has shown inconsistent results about potential relationships between anger, disgust, and anxiety with misophonia (Schröder et al., 2013; McKay et al., 2018; Jager et al., 2020). In our study, both anger and disgust were significantly and positively associated with the two summary misophonia scores (weighted and unweighted) over time. Assessing the individual elements of misophonia, disgust was significantly and positively associated with the emotional misophonic response and perceptions of participation in life, with stronger feelings of disgust at baseline predicting a stronger misophonic response over time. Our results therefore support studies showing disgust is associated with misophonia (Edelstein et al., 2013; Schröder et al., 2013; Rouw and Erfanian, 2018) but contradict other research showing disgust is not associated with misophonia (Jager et al., 2020). Similarly, the stronger the feelings of anger, the longer the recovery time after the misophonic response and the stronger the physiological response to the trigger. This supports both qualitative and quantitative studies that have reported anger in people with misophonia (Edelstein et al., 2013; Schröder et al., 2013; Wu et al., 2014; McKay et al., 2018; Rouw and Erfanian, 2018; Jager et al., 2020).

Taken together, our longitudinal results indicate that disgust is associated with more of an emotional response while anger is associated with more of a physiological response, which supports previous cross-sectional findings (Edelstein et al., 2013; Schröder et al., 2013; Wu et al., 2014; McKay et al., 2018; Rouw and Erfanian, 2018). That anger and disgust were associated with different aspects of the misophonic response may explain previous contradictory findings regarding which of these two emotions is important. It seems possible that a measure of misophonia which includes more emotional items may show disgust to be the stronger emotion, while those which include the physiological responses may show anger as the dominant emotion.

Anxiety has also been proposed as an important aspect of misophonia, although again the literature is contradictory (Schröder et al., 2013; Taylor, 2017; McKay et al., 2018). Our results support those studies that propose anxiety is not the primary emotion (Taylor, 2017; Jager et al., 2020) as anxiety did not emerge as a significant predictor of misophonic responses. Anxiety was only significantly predictive of one aspect of misophonia, avoidance of the trigger, but this relationship lost significance once depression was included. Overall, our results indicate the importance of anger and disgust over anxiety in relation to the misophonic response.

### Quality of life and misophonia

Another of our aims was to determine perceptions of quality of life in those with self-reported misophonia. Using the RAND Corporation version of the SF-36 (Ware and Sherbourne, 1992) to measure quality of life, which has been widely used across many different physical and mental health conditions, allowed comparison of quality of life in those with self-reported misophonia in our sample to quality of life reported in different groups in other studies. Relative to the general population in our sample, there were significantly lower ratings on all domains of quality of life for our participants with self-reported misophonia. In addition, compared to those with long-term conditions (Bowling et al., 1999), our misophonia group scored lower on the ability to carry out their role due to emotional problems, lower emotional wellbeing, lower social functioning and greater fatigue levels. The quality of life scores for those with misophonia were higher than people with OCD (Rodriguez-Salgado et al., 2006) but were lower than those with tinnitus (Ross et al., 2007) for energy, social functioning and role limitations due to emotional problems. This shows that relative to those with other conditions (Rodriguez-Salgado et al., 2006; Ross et al., 2007) and those without misophonia (Bowling et al., 1999; Rodriguez-Salgado et al., 2006) the misophonic experience interrupts the individual’s abilities to perform their role or task and engage in their social life. The impact on social functioning is likely due to the role that others play in triggering the individual with misophonia, however, further research would be able to explore this. These results also make it clear that, while misophonia is not yet recognized, it is nonetheless impacting quality of life. The two lowest scoring domains (role limitations due to emotional problems and energy/fatigue) show where the main impact is for people with misophonia.

In addition, there were no significant changes over time in quality of life for people in our study with self-reported misophonia, whereas the pattern for the general population group in our study showed a decrease over time on two subscales (energy/fatigue and emotional wellbeing). This may be an effect of ‘lockdown’ as our data were collected during the first 18 months of the COVID-19 pandemic, however, as no measures were taken in relation to lockdowns we cannot say for certain.

### Does misophonia predict quality of life over time?

With regard to understanding the role misophonia plays in perceiving quality of life, cross-sectionally, misophonia was associated with quality of life, however, many of these associations weakened or ceased to be significant once anxiety, self-esteem and depression were included. Longitudinally, misophonic responses were predictive of quality of life, but only on two of the domains. Greater role limitations due to emotional problems were predicted by less avoidance of triggers, a stronger emotional response to the trigger, and perceptions of more impact on participation in life. Greater pain was predicted by a greater perception of impact on participation in life. These relationships remained significant even after depression was included. A third domain of quality of life was also predicted by misophonia, with a stronger emotional response to triggers predicting greater fatigue; however, this relationship ceased to be significant once depression was entered, again showing the strong effects of depression on quality of life. These results partially support qualitative reports where some participants reported great impact (and others less so) (Edelstein et al., 2013) and quantitative results showing “slightly lower quality of life” ratings for people with misophonia in a cross-sectional study (Jager et al., 2020, p. 8).

The importance of depression across all the quality of life domains cross-sectionally, and four domains longitudinally, shows the strong impact of depressive symptoms on quality of life. There was evidence of some mediation as some misophonic factors were no longer significant once depression was entered into the regression models. This supports many quality of life studies that have demonstrated the impact of depression on quality of life (for example, Friedman et al., 2005).

Despite the effects of depression, misophonia was important for all domains of quality of life, cross-sectionally. Longitudinally, emotional response to triggers, avoidance of triggers and perceived impact of participation, remained predictive of role limitations due to emotional problems, while perceived impact of participation was predictive of perceptions of pain. This demonstrates the potential long-term impact of some aspects of the experience of misophonia on perceptions of quality of life. In particular, our findings highlight that (at least for this sample) the emotional aspects of the misophonic response, as well as the need to avoid triggers and feeling like the ability to participate in life is reduced, appear to have a greater impact on quality of life than does the physiological aspect of the misophonic response, or the length of time taken to recover or the frequency of being triggered.

### Limitations and future directions

The recruitment strategy is one limitation of this study. Participants were recruited online via social media and through the Misophonia Institute, United States, which means that people with misophonia who are not associated with this organization, or anyone who does not use social media, would not have been able to participate, meaning we cannot be certain that the results are relevant to all people with misophonia. A current limitation of many studies in this field relates to a lack of being able to receive a formal diagnosis of misophonia in most countries, which means we cannot be sure that all our participants actually had misophonia (as participants self-reported whether they considered themselves to have misophonia or not in response to a single-item question, rather than any formal diagnosis process as part of the study).

Another limitation lies in that while self-reported co-morbid conditions were recorded, these data were not included in the statistical analysis as assessing the incidence of co-morbid conditions alongside misophonia was beyond the scope of this study; these items were included to help descriptively assess the study sample at baseline only. Furthermore, we did not collect any data regarding participants’ psychological or psychiatric histories regarding previous and existing conditions and/or treatments. We therefore cannot rule out the possibility that some people who stated they had misophonia may in fact have been experiencing sensory triggers as a result of other co-morbid or undiagnosed psychiatric conditions. Indeed, one study recently identified that 26% of patients referred with a self-diagnosis of misophonia were deemed by psychiatrics to have a different primary condition (Jager et al., 2020). Future research should aim to recruit clinical samples, individuals with links to other misophonia organizations, and those who do not engage with social media.

Data were collected during the COVID-19 pandemic, which may have impacted exposure to triggers and quality of life for all participants, but as countries responded differently and at different times to the spread of the virus, it is difficult to know the effects of the pandemic and associated lockdowns on the data. Our sample was also predominantly female and future research should aim to recruit more men. Our longitudinal study was conducted over a period of 6 months, which is a strength; however, as is common with longitudinal studies we expected, and observed, a high attrition rate (Boys et al., 2003; Gustavson et al., 2012). Longitudinal research is important in order to understand the long-term effects of misophonia over a longer period, to explore how misophonia may change and progress over time. Finally, three work-related items were included in this research, yielding interesting results in terms of perceptions of noise levels at work. Future research into how the work environment influences work satisfaction would benefit those living with misophonia.

## Conclusion

This research has shown that people with self-reported misophonia rate their quality of life as lower than those who have tinnitus and much lower than people without misophonia and without other long-term physical conditions. This shows how this condition does indeed impact on quality of life in a significant way. Anger and disgust were found to be the main negative emotions associated with misophonia, while anxiety was not associated with misophonia. The strong influence of depression with both cross-sectional and longitudinal analysis, suggests that treatment for misophonia could include strategies to tackle depression as part of the treatment. Misophonia was associated with perceptions of quality of life over time, in particular, the emotional response to the trigger and the perceived impact on participation in life were the main factors associated with the ability to carry out one’s role and the perception of pain. These results show the impact misophonia can have and highlight this condition as one that needs further research and support.

## Data availability statement

The raw data supporting the conclusions of this article will be made available by the authors, without undue reservation.

## Ethics statement

This research received ethical approval from the University of Surrey Ethics Committee. All participants provided informed consent to participate.

## Author contributions

BD designed the study. Both authors were involved in the data collection, analysis, and write up of the study and approved the submitted version.

## Conflict of interest

The authors declare that the research was conducted in the absence of any commercial or financial relationships that could be construed as a potential conflict of interest.

## Publisher’s note

All claims expressed in this article are solely those of the authors and do not necessarily represent those of their affiliated organizations, or those of the publisher, the editors and the reviewers. Any product that may be evaluated in this article, or claim that may be made by its manufacturer, is not guaranteed or endorsed by the publisher.

## Footnotes

1 A subset of the data was published in Dibb et al. (2021).

2 Medical Outcomes Study SF-36 available from RAND Corporation here: https://www.rand.org/health-care/surveys_tools/mos/36-item-short-form.html

3 Distributions for some outcome variables were skewed (MRS scores in the general population and quality of life scores for both groups); given the large sample sizes achieved, parametric tests were performed and reported for tests of difference between groups at baseline and follow-up, and within-participants over time. As a sense check, non-parametric tests were also performed; patterns of statistical significance achieved (i.e., significant or not) were the same for all variables, with one exception; the significant difference observed in physical functioning on the SF-36 between groups at baseline using an independent *t*-test, was not statistically significant on the Mann–Whitney test (*p* = 0.13).

4 Assumptions for independence of errors and multicollinearity were met for all models. Standardized residuals indicated a small percentage of cases (<1%) represented multivariate outliers in some models; values for leverage and Mahalanobis’ distance indicated these cases may exert some undue influence in the models. Overall, however, the assumptions for regression models were met; to increase the robustness of all estimates, models were bootstrapped.

5 Belgium, Botswana, Brazil, Colombia, Denmark, France, Germany, Greece, Italy, Netherlands, Norway, Peru, Spain, Switzerland, and Thailand.
