# Corrigendum: A longitudinal investigation of quality of life and negative emotions in misophonia

**DOI:** 10.3389/fnins.2023.1266908

**Published:** 2023-11-15

**Authors:** Bridget Dibb, Sarah E. Golding

**Affiliations:** School of Psychology, Faculty of Health and Medical Sciences, University of Surrey, Guildford, United Kingdom

**Keywords:** misophonia, quality of life, misophonia response scale, anger, disgust, anxiety, depression

In the article, there was an error. In the discussion the word “greater” appears when it should read “less”.

A correction has been made to **Section: Discussion**, *Subsection:* Does misophonia predict quality of life over time?, Paragraph number: 1.

This sentence previously stated:

“Greater role limitations due to emotional problems were predicted by greater avoidance of triggers, a stronger emotional response to the trigger, and perceptions of more impact on participation in life.”

The corrected sentence appears below:

“Greater role limitations due to emotional problems were predicted by less avoidance of triggers, a stronger emotional response to the trigger, and perceptions of more impact on participation in life.”

The authors apologize for this error and state that this does not change the scientific conclusions of the article in any way. The original article has been updated.

