# Supplementary material for: A longitudinal investigation of quality of life and negative emotions in misophonia
Source: Front Neurosci. 2022 Jul 22;16:900474. doi: 10.3389/fnins.2022.900474 (PMC9354518; doi:10.3389/fnins.2022.900474)
Supplement: Supplementary file 1 [file Table_1.docx]

**Appendix**

**Full Regression Tables for Predicting Misophonic Responses and Quality of Life**

# Table A.1 - Hierarchical Regression Models for Predicting Misophonic Responses (Frequency) in Misophonia Group at Baseline and Follow-Up

|  | ***B* [BCa 95% CI]** | ***SE B*** | ***β*** | ***t*** | ***p*** | ***F* (df)** | ***p*** | ***R^2^*** | **Δ*R^2^*** | ***p*** |
| --- | --- | --- | --- | --- | --- | --- | --- | --- | --- | --- |
| **BASELINE** |  |  |  |  |  |  |  |  |  |  |
| **Block 1** |  |  |  |  |  | 22.81 (1,489) | < .001 | .05 |  |  |
| Constant | 4.81 | 0.18 |  | 26.36 | < .001 |  |  |  |  |  |
| Age | -0.01 | < 0.01 | -.21 | 4.78 | < .001 |  |  |  |  |  |
| **Block 2** |  |  |  |  |  | 28.35 (4,486) | < .001 |  | .15 | < .001 |
| Constant | 2.14 | 0.34 |  | 6.33 | < .001 |  |  |  |  |  |
| Age | -0.01 | < 0.01 | -.14 | 3.22 | .001 |  |  |  |  |  |
| Anxiety | 0.20 | 0.08 | .12 | 2.54 | .012 |  |  |  |  |  |
| Anger | 0.08 | 0.09 | .05 | 0.97 | .33 |  |  |  |  |  |
| Disgust | 0.53 | 0.08 | .31 | 6.92 | < .001 |  |  |  |  |  |
| **Block 3** |  |  |  |  |  | 24.37 (5,485) | < .001 |  | .01 | .008 |
| Constant | 2.00 | 0.34 |  | 5.87 | < .001 |  |  |  |  |  |
| Age | -0.01 | < 0.01 | -.13 | 3.18 | .002 |  |  |  |  |  |
| Anxiety | 0.04 | 0.10 | .02 | 0.37 | .72 |  |  |  |  |  |
| Anger | 0.05 | 0.09 | .03 | 0.53 | .59 |  |  |  |  |  |
| Disgust | 0.50 | 0.08 | .29 | 6.37 | < .001 |  |  |  |  |  |
| Depression | 0.32 | 0.12 | .16 | 2.66 | .008 |  |  |  |  |  |
| **FOLLOW-UP** |  |  |  |  |  |  |  |  |  |  |
| **Block 1** |  |  |  |  |  | 3.04 (1,125) | .084 | .02 |  |  |
| Constant | 4.56 | 0.40 |  | 11.39 | < .001 |  |  |  |  |  |
| Age | -0.01 | 0.01 | -.15 | 1.74 | .084 |  |  |  |  |  |
| **Block 2** |  |  |  |  |  | 4.23 (4,122) | .003 |  | .10 | .004 |
| Constant | 1.98 | 0.80 |  | 2.46 | .015 |  |  |  |  |  |
| Age | -0.01 | 0.01 | -.11 | 1.27 | .21 |  |  |  |  |  |
| Anxiety | 0.31 | 0.19 | .15 | 1.65 | .10 |  |  |  |  |  |
| Anger | 0.24 | 0.20 | .12 | 1.21 | .23 |  |  |  |  |  |
| Disgust | 0.35 | 0.18 | .18 | 1.93 | .056 |  |  |  |  |  |
| **Block 3** |  |  |  |  |  | 3.64 (5,121) | .004 |  | .01 | .30 |
| Constant | 1.83 | 0.82 |  | 2.24 | .027 |  |  |  |  |  |
| Age | -0.01 | 0.01 | -.10 | 1.14 | .26 |  |  |  |  |  |
| Anxiety | 0.15 | 0.24 | .08 | 0.63 | .53 |  |  |  |  |  |
| Anger | 0.19 | 0.20 | .09 | 0.94 | .35 |  |  |  |  |  |
| Disgust | 0.31 | 0.19 | .15 | 1.67 | .099 |  |  |  |  |  |
| Depression | 0.31 | 0.30 | .13 | 1.04 | .30 |  |  |  |  |  |

*Note.* Bootstrap results are based on 1,000 bootstrap samples. BCa 95% CI = 95% bias-corrected and accelerated bootstrap confidence intervals. df = degrees of freedom.

# Table A.2 - Hierarchical Regression Models for Predicting Misophonic Responses (Recovery) in Misophonia Group at Baseline and Follow-Up

|  | ***B* [BCa 95% CI]** | ***SE B*** | ***β*** | ***t*** | ***p*** | ***F* (df)** | ***p*** | ***R^2^*** | **Δ*R^2^*** | ***p*** |
| --- | --- | --- | --- | --- | --- | --- | --- | --- | --- | --- |
| **BASELINE** |  |  |  |  |  |  |  |  |  |  |
| **Block 1** |  |  |  |  |  | 7.58 (1,489) | .006 | .02 |  |  |
| Constant | 4.56 | 0.19 |  | 23.66 | < .001 |  |  |  |  |  |
| Age | -0.01 | < 0.01 | -.12 | 2.75 | .006 |  |  |  |  |  |
| **Block 2** |  |  |  |  |  | 21.80 (4,486) | < .001 |  | .14 | < .001 |
| Constant | 2.00 | 0.36 |  | 5.44 | < .001 |  |  |  |  |  |
| Age | < -0.01 | < 0.01 | -.05 | 1.12 | .27 |  |  |  |  |  |
| Anxiety | 0.31 | 0.09 | .17 | 3.70 | < .001 |  |  |  |  |  |
| Anger | 0.35 | 0.09 | .19 | 3.88 | < .001 |  |  |  |  |  |
| Disgust | 0.22 | 0.08 | .12 | 2.66 | .008 |  |  |  |  |  |
| **Block 3** |  |  |  |  |  | 18.05 (5,485) | < .001 |  | .005 | .098 |
| Constant | 1.86 | 0.36 |  | 5.12 | < .001 |  |  |  |  |  |
| Age | < -0.01 | < 0.01 | -.05 | 1.08 | .28 |  |  |  |  |  |
| Anxiety | 0.20 | 0.12 | .11 | 1.89 | .059 |  |  |  |  |  |
| Anger | 0.33 | 0.09 | .18 | 3.57 | < .001 |  |  |  |  |  |
| Disgust | 0.19 | 0.08 | .11 | 2.32 | .021 |  |  |  |  |  |
| Depression | 0.22 | 0.13 | .10 | 1.66 | .098 |  |  |  |  |  |
| **FOLLOW-UP** |  |  |  |  |  |  |  |  |  |  |
| **Block 1** |  |  |  |  |  | 0.80 (1,125) | .37 | < .01 |  |  |
| Constant | 4.22 | 0.38 |  | 10.98 | < .001 |  |  |  |  |  |
| Age | -0.01 | 0.01 | -.08 | 0.89 | .37 |  |  |  |  |  |
| **Block 2** |  |  |  |  |  | 4.27 (4,122) | .003 |  | .12 | .002 |
| Constant | 2.09 | 0.76 |  | 2.74 | .007 |  |  |  |  |  |
| Age | < -0.01 | 0.01 | -.05 | 0.59 | .56 |  |  |  |  |  |
| Anxiety | 0.01 | 0.18 | .004 | 0.04 | .97 |  |  |  |  |  |
| Anger | 0.57 | 0.19 | .29 | 3.00 | .003 |  |  |  |  |  |
| Disgust | 0.21 | 0.17 | .11 | 1.20 | .23 |  |  |  |  |  |
| **Block 3** |  |  |  |  |  | 3.91 (5,121) | .003 |  | .02 | .13 |
| Constant | 1.88 | 0.77 |  | 2.44 | .016 |  |  |  |  |  |
| Age | < -0.01 | 0.01 | -.04 | 0.41 | .68 |  |  |  |  |  |
| Anxiety | -0.21 | 0.23 | -.11 | 0.93 | .36 |  |  |  |  |  |
| Anger | 0.50 | 0.19 | .26 | 2.59 | .011 |  |  |  |  |  |
| Disgust | 0.15 | 0.18 | .08 | 0.85 | .40 |  |  |  |  |  |
| Depression | 0.43 | 0.28 | .19 | 1.52 | .13 |  |  |  |  |  |

*Note.* Bootstrap results are based on 1,000 bootstrap samples. BCa 95% CI = 95% bias-corrected and accelerated bootstrap confidence intervals. df = degrees of freedom.

# Table A.3 - Hierarchical Regression Models for Predicting Misophonic Responses (Avoidance) in Misophonia Group at Baseline and Follow-Up

|  | ***B* [BCa 95% CI]** | ***SE B*** | ***β*** | ***t*** | ***p*** | ***F* (df)** | ***p*** | ***R^2^*** | **Δ*R^2^*** | ***p*** |
| --- | --- | --- | --- | --- | --- | --- | --- | --- | --- | --- |
| **BASELINE** |  |  |  |  |  |  |  |  |  |  |
| **Block 1** |  |  |  |  |  | < .01 (1,489) | .98 | < .01 |  |  |
| Constant | 5.47 | 0.24 |  | 22.97 | < .001 |  |  |  |  |  |
| Age | < 0.01 | < 0.01 | < -.01 | 0.03 | .98 |  |  |  |  |  |
| **Block 2** |  |  |  |  |  | 6.77 (4,486) | < .001 |  | .05 | < .001 |
| Constant | 3.53 | 0.47 |  | 7.57 | < .001 |  |  |  |  |  |
| Age | < 0.01 | < 0.01 | .05 | 1.06 | .29 |  |  |  |  |  |
| Anxiety | -0.03 | 0.11 | -.01 | 0.24 | .81 |  |  |  |  |  |
| Anger | 0.34 | 0.12 | .15 | 2.93 | .004 |  |  |  |  |  |
| Disgust | 0.29 | 0.12 | .13 | 2.75 | .006 |  |  |  |  |  |
| **Block 3** |  |  |  |  |  | 7.47 (5,485) | < .001 |  | .02 | .002 |
| Constant | 3.30 | 0.47 |  | 7.05 | < .001 |  |  |  |  |  |
| Age | < 0.01 | < 0.01 | .05 | 1.15 | .25 |  |  |  |  |  |
| Anxiety | -0.30 | 0.14 | -.13 | 2.12 | .034 |  |  |  |  |  |
| Anger | 0.28 | 0.12 | .13 | 2.41 | .017 |  |  |  |  |  |
| Disgust | 0.23 | 0.12 | .11 | 2.17 | .031 |  |  |  |  |  |
| Depression | 0.53 | 0.17 | .20 | 3.12 | .002 |  |  |  |  |  |
| **FOLLOW-UP** |  |  |  |  |  |  |  |  |  |  |
| **Block 1** |  |  |  |  |  | 0.03 (1,125) | .88 | < .01 |  |  |
| Constant | 5.46 | 0.49 |  | 11.14 | < .001 |  |  |  |  |  |
| Age | < 0.01 | 0.01 | .01 | 0.16 | .88 |  |  |  |  |  |
| **Block 2** |  |  |  |  |  | 2.07 (4,122) | .089 |  | .06 | .045 |
| Constant | 3.93 | 1.00 |  | 3.92 | < .001 |  |  |  |  |  |
| Age | < 0.01 | 0.01 | .02 | 0.24 | .81 |  |  |  |  |  |
| Anxiety | -0.27 | 0.23 | -.11 | 1.15 | .25 |  |  |  |  |  |
| Anger | 0.50 | 0.25 | .20 | 2.00 | .048 |  |  |  |  |  |
| Disgust | 0.30 | 0.23 | .12 | 1.33 | .19 |  |  |  |  |  |
| **Block 3** |  |  |  |  |  | 1.73 (4.64) | .13 |  | .003 | .52 |
| Constant | 4.05 | 1.02 |  | 3.96 | < .001 |  |  |  |  |  |
| Age | < 0.01 | 0.01 | .02 | 0.17 | .87 |  |  |  |  |  |
| Anxiety | -0.15 | 0.30 | -.06 | 0.49 | .63 |  |  |  |  |  |
| Anger | 0.53 | 0.26 | .21 | 2.09 | .039 |  |  |  |  |  |
| Disgust | 0.33 | 0.23 | .14 | 1.43 | .15 |  |  |  |  |  |
| Depression | -0.24 | 0.38 | -.08 | 0.64 | .52 |  |  |  |  |  |

*Note.* Bootstrap results are based on 1,000 bootstrap samples. BCa 95% CI = 95% bias-corrected and accelerated bootstrap confidence intervals. df = degrees of freedom.

# Table A.4 - Hierarchical Regression Models for Predicting Misophonic Responses (Emotional Responses) in Misophonia Group at Baseline and Follow-Up

|  | ***B* [BCa 95% CI]** | ***SE B*** | ***β*** | ***t*** | ***p*** | ***F* (df)** | ***p*** | ***R^2^*** | **Δ*R^2^*** | ***p*** |
| --- | --- | --- | --- | --- | --- | --- | --- | --- | --- | --- |
| **BASELINE** |  |  |  |  |  |  |  |  |  |  |
| **Block 1** |  |  |  |  |  | 26.59 (1,489) | < .001 | .05 |  |  |
| Constant | 6.43 | 0.16 |  | 40.12 | < .001 |  |  |  |  |  |
| Age | -0.02 | < 0.01 | -.23 | 5.16 | < .001 |  |  |  |  |  |
| **Block 2** |  |  |  |  |  | 32.93 (4,486) | < .001 |  | .16 | < .001 |
| Constant | 3.92 | 0.29 |  | 13.35 | < .001 |  |  |  |  |  |
| Age | -0.01 | < 0.01 | -.14 | 3.46 | .001 |  |  |  |  |  |
| Anxiety | 0.12 | 0.07 | .08 | 1.72 | .086 |  |  |  |  |  |
| Anger | 0.19 | 0.07 | .12 | 2.58 | .010 |  |  |  |  |  |
| Disgust | 0.46 | 0.07 | .31 | 6.95 | < .001 |  |  |  |  |  |
| **Block 3** |  |  |  |  |  | 26.95 (5,485) | < .001 |  | .004 | .11 |
| Constant | 3.84 | 0.30 |  | 12.95 | < .001 |  |  |  |  |  |
| Age | -0.01 | < 0.01 | -.14 | 3.42 | .001 |  |  |  |  |  |
| Anxiety | 0.03 | 0.09 | .02 | 0.35 | .72 |  |  |  |  |  |
| Anger | 0.17 | 0.08 | .11 | 2.28 | .023 |  |  |  |  |  |
| Disgust | 0.44 | 0.07 | .30 | 6.56 | < .001 |  |  |  |  |  |
| Depression | 0.17 | 0.12 | .10 | 1.62 | .11 |  |  |  |  |  |
| **FOLLOW-UP** |  |  |  |  |  |  |  |  |  |  |
| **Block 1** |  |  |  |  |  | 6.73 (1,125) | .011 | .05 |  |  |
| Constant | 6.23 | 0.36 |  | 17.34 | < .001 |  |  |  |  |  |
| Age | -0.02 | 0.01 | -.23 | 2.59 | .011 |  |  |  |  |  |
| **Block 2** |  |  |  |  |  | 5.23 (4,122) | .001 |  | .10 | .005 |
| Constant | 4.52 | 0.73 |  | 6.21 | < .001 |  |  |  |  |  |
| Age | -0.02 | 0.01 | -.21 | 2.45 | .016 |  |  |  |  |  |
| Anxiety | -0.15 | 0.17 | -.08 | 0.88 | .38 |  |  |  |  |  |
| Anger | 0.35 | 0.18 | .19 | 1.95 | .054 |  |  |  |  |  |
| Disgust | 0.39 | 0.16 | .21 | 2.37 | .019 |  |  |  |  |  |
| **Block 3** |  |  |  |  |  | 4.15 (5,121) | .002 |  | < .01 | .97 |
| Constant | 4.53 | 0.74 |  | 6.09 | < .001 |  |  |  |  |  |
| Age | -0.02 | 0.01 | -.21 | 2.43 | .017 |  |  |  |  |  |
| Anxiety | -0.14 | 0.22 | -.08 | 0.65 | .51 |  |  |  |  |  |
| Anger | 0.35 | 0.19 | .19 | 1.90 | .060 |  |  |  |  |  |
| Disgust | 0.39 | 0.17 | .21 | 2.31 | .022 |  |  |  |  |  |
| Depression | -0.01 | 0.27 | -.01 | 0.04 | .97 |  |  |  |  |  |

*Note.* Bootstrap results are based on 1,000 bootstrap samples. BCa 95% CI = 95% bias-corrected and accelerated bootstrap confidence intervals. df = degrees of freedom.

# Table A.5 - Hierarchical Regression Models for Predicting Misophonic Responses (Physiological Responses) in Misophonia Group at Baseline and Follow-Up

|  | ***B* [BCa 95% CI]** | ***SE B*** | ***β*** | ***t*** | ***p*** | ***F* (df)** | ***p*** | ***R^2^*** | **Δ*R^2^*** | ***p*** |
| --- | --- | --- | --- | --- | --- | --- | --- | --- | --- | --- |
| **BASELINE** |  |  |  |  |  |  |  |  |  |  |
| **Block 1** |  |  |  |  |  | 20.99 (1,489) | < .001 | .04 |  |  |
| Constant | 4.09 | 0.20 |  | 20.91 | < .001 |  |  |  |  |  |
| Age | -0.02 | < 0.01 | 0.20 | 4.60 | < .001 |  |  |  |  |  |
| **Block 2** |  |  |  |  |  | 34.44 (4,486) | < .001 |  | .18 | < .001 |
| Constant | 0.95 | 0.36 |  | 2.67 | .008 |  |  |  |  |  |
| Age | -0.01 | < 0.01 | -.11 | 2.76 | .006 |  |  |  |  |  |
| Anxiety | 0.30 | 0.08 | .16 | 3.56 | < .001 |  |  |  |  |  |
| Anger | 0.42 | 0.09 | .23 | 4.76 | < .001 |  |  |  |  |  |
| Disgust | 0.32 | 0.08 | .18 | 4.00 | < .001 |  |  |  |  |  |
| **Block 3** |  |  |  |  |  | 29.00 (5,485) | < .001 |  | .01 | .016 |
| Constant | 0.81 | 0.36 |  | 2.27 | .024 |  |  |  |  |  |
| Age | -0.01 | < 0.01 | -.11 | 2.72 | .007 |  |  |  |  |  |
| Anxiety | 0.14 | 0.12 | .08 | 1.32 | .19 |  |  |  |  |  |
| Anger | 0.39 | 0.09 | .21 | 4.32 | < .001 |  |  |  |  |  |
| Disgust | 0.29 | 0.08 | .16 | 3.52 | < .001 |  |  |  |  |  |
| Depression | 0.31 | 0.13 | .14 | 2.42 | .016 |  |  |  |  |  |
| **FOLLOW-UP** |  |  |  |  |  |  |  |  |  |  |
| **Block 1** |  |  |  |  |  | 15,24 (1,125) | < .001 | .11 |  |  |
| Constant | 4.57 | 0.41 |  | 11.13 | < .001 |  |  |  |  |  |
| Age | -0.03 | 0.01 | -.33 | 3.90 | < .001 |  |  |  |  |  |
| **Block 2** |  |  |  |  |  | 8.18 (4,122) | < .001 |  | .10 | .002 |
| Constant | 2.07 | 0.82 |  | 2.53 | .013 |  |  |  |  |  |
| Age | -0.03 | 0.01 | -.30 | 3.65 | < .001 |  |  |  |  |  |
| Anxiety | 0.07 | 0.19 | .031 | 0.35 | .73 |  |  |  |  |  |
| Anger | 0.51 | 0.20 | .23 | 2.51 | .013 |  |  |  |  |  |
| Disgust | 0.31 | 0.18 | .15 | 1.70 | .091 |  |  |  |  |  |
| **Block 3** |  |  |  |  |  | 12.27 (5,121) | < .001 |  | .01 | .16 |
| Constant | 1.86 | 0.83 |  | 2.25 | .026 |  |  |  |  |  |
| Age | -0.03 | 0.01 | -.28 | 3.48 | .001 |  |  |  |  |  |
| Anxiety | -0.15 | 0.24 | -.07 | 0.61 | .54 |  |  |  |  |  |
| Anger | 0.44 | 0.21 | .20 | 2.13 | .035 |  |  |  |  |  |
| Disgust | 0.26 | 0.19 | .12 | 1.37 | .18 |  |  |  |  |  |
| Depression | 0.43 | 0.30 | .17 | 1.40 | .16 |  |  |  |  |  |

*Note.* Bootstrap results are based on 1,000 bootstrap samples. BCa 95% CI = 95% bias-corrected and accelerated bootstrap confidence intervals. df = degrees of freedom.

# Table A.6 - Hierarchical Regression Models for Predicting Misophonic Responses (Participation in Life) in Misophonia Group at Baseline and Follow-Up

|  | ***B* [BCa 95% CI]** | ***SE B*** | ***β*** | ***t*** | ***p*** | ***F* (df)** | ***p*** | ***R^2^*** | **Δ*R^2^*** | ***p*** |
| --- | --- | --- | --- | --- | --- | --- | --- | --- | --- | --- |
| **BASELINE** |  |  |  |  |  |  |  |  |  |  |
| **Block 1** |  |  |  |  |  | 5.76 (1,489) | .017 | .01 |  |  |
| Constant | 4.64 | 0.21 |  | 22.10 | < .001 |  |  |  |  |  |
| Age | -0.01 | < 0.01 | -.11 | 2.40 | .017 |  |  |  |  |  |
| **Block 2** |  |  |  |  |  | 37.04 (4,486) | < .001 |  | .22 | < .001 |
| Constant | 0.91 | 0.37 |  | 2.44 | .015 |  |  |  |  |  |
| Age | < -0.01 | < 0.01 | -.01 | 0.18 | .86 |  |  |  |  |  |
| Anxiety | 0.32 | 0.09 | .16 | 3.64 | < .001 |  |  |  |  |  |
| Anger | 0.48 | 0.09 | .24 | 5.17 | < .001 |  |  |  |  |  |
| Disgust | 0.42 | 0.08 | .22 | 5.03 | < .001 |  |  |  |  |  |
| **Block 3** |  |  |  |  |  | 34.24 (5,485) | < .001 |  | .03 | < .001 |
| Constant | 0.66 | 0.37 |  | 1.78 | .076 |  |  |  |  |  |
| Age | < 0.01 | < 0.01 | < -.01 | 0.08 | .94 |  |  |  |  |  |
| Anxiety | 0.03 | 0.11 | .02 | 0.30 | .77 |  |  |  |  |  |
| Anger | 0.42 | 0.09 | .21 | 4.50 | < .001 |  |  |  |  |  |
| Disgust | 0.36 | 0.08 | .19 | 4.28 | < .001 |  |  |  |  |  |
| Depression | 0.56 | 0.13 | .24 | 4.23 | < .001 |  |  |  |  |  |
| **FOLLOW-UP** |  |  |  |  |  |  |  |  |  |  |
| **Block 1** |  |  |  |  |  | 4.59 (1,125) | .034 | .04 |  |  |
| Constant | 4.88 | 0.42 |  | 11.72 | < .001 |  |  |  |  |  |
| Age | -0.02 | 0.01 | -.19 | 2.14 | .034 |  |  |  |  |  |
| **Block 2** |  |  |  |  |  | 7.68 (4,122) | < .001 |  | .17 | < .001 |
| Constant | 1.48 | 0.80 |  | 1.85 | .067 |  |  |  |  |  |
| Age | -0.01 | 0.01 | -.14 | 1.70 | .092 |  |  |  |  |  |
| Anxiety | 0.20 | 0.19 | .09 | 1.07 | .29 |  |  |  |  |  |
| Anger | 0.40 | 0.20 | .19 | 2.02 | .045 |  |  |  |  |  |
| Disgust | 0.56 | 0.18 | .27 | 3.10 | .002 |  |  |  |  |  |
| **Block 3** |  |  |  |  |  | 6.24 (5,121) | < .001 |  | .004 | .44 |
| Constant | 1.37 | 0.82 |  | 1.68 | .097 |  |  |  |  |  |
| Age | -0.01 | 0.01 | -.13 | 1.60 | .11 |  |  |  |  |  |
| Anxiety | 0.08 | 0.24 | .04 | 0.34 | .74 |  |  |  |  |  |
| Anger | 0.37 | 0.20 | .17 | 1.79 | .076 |  |  |  |  |  |
| Disgust | 0.53 | 0.19 | .25 | 2.85 | .005 |  |  |  |  |  |
| Depression | 0.23 | 0.30 | .09 | 0.78 | .44 |  |  |  |  |  |

*Note.* Bootstrap results are based on 1,000 bootstrap samples. BCa 95% CI = 95% bias-corrected and accelerated bootstrap confidence intervals. df = degrees of freedom.

# Table A.7 - Hierarchical Regression Models for Predicting Misophonic Responses (Severity Score) in Misophonia Group at Baseline and Follow-Up

|  | ***B* [BCa 95% CI]** | ***SE B*** | ***β*** | ***t*** | ***p*** | ***F* (df)** | ***p*** | ***R^2^*** | **Δ*R^2^*** | ***p*** |
| --- | --- | --- | --- | --- | --- | --- | --- | --- | --- | --- |
| **BASELINE** |  |  |  |  |  |  |  |  |  |  |
| **Block 1** |  |  |  |  |  | 26.17 (1,489) | < .001 | .05 |  |  |
| Constant | 5.10 | 0.15 |  | 33.98 | < .001 |  |  |  |  |  |
| Age | -0.02 | < 0.01 | -.23 | 5.12 | < .001 |  |  |  |  |  |
| **Block 2** |  |  |  |  |  | 44.81 (4,486) | < .001 |  | .27 | < .001 |
| Constant | 2.03 | 0.25 |  | 7.99 | < .001 |  |  |  |  |  |
| Age | -0.01 | < 0.01 | -.11 | 2.96 | .003 |  |  |  |  |  |
| Anxiety | 0.24 | 0.06 | .17 | 3.96 | < .001 |  |  |  |  |  |
| Anger | 0.35 | 0.06 | .24 | 5.53 | < .001 |  |  |  |  |  |
| Disgust | 0.40 | 0.06 | .29 | 6.94 | < .001 |  |  |  |  |  |
| **Block 3** |  |  |  |  |  | 50.49 (5,485) | < .001 |  | .02 | < .001 |
| Constant | 1.89 | 0.25 |  | 7.41 | < .001 |  |  |  |  |  |
| Age | -0.01 | < 0.01 | -.11 | 2.91 | .004 |  |  |  |  |  |
| Anxiety | 0.07 | 0.08 | .05 | 0.95 | .34 |  |  |  |  |  |
| Anger | 0.32 | 0.06 | .22 | 4.94 | < .001 |  |  |  |  |  |
| Disgust | 0.36 | 0.06 | .26 | 6.28 | < .001 |  |  |  |  |  |
| Depression | 0.33 | 0.09 | .19 | 3.57 | < .001 |  |  |  |  |  |
| **FOLLOW-UP** |  |  |  |  |  |  |  |  |  |  |
| **Block 1** |  |  |  |  |  | 14.17 (1,125) | < .001 | .10 |  |  |
| Constant | 5.29 | 0.31 |  | 16.96 | < .001 |  |  |  |  |  |
| Age | -0.02 | 0.01 | -.31 | 3.76 | < .001 |  |  |  |  |  |
| **Block 2** |  |  |  |  |  | 11.41 (1,122) | < .001 |  | .17 | < .001 |
| Constant | 2.82 | 0.59 |  | 4.75 | < .001 |  |  |  |  |  |
| Age | -0.02 | 0.01 | -.28 | 3.57 | .001 |  |  |  |  |  |
| Anxiety | 0.02 | 0.14 | .01 | 0.16 | .87 |  |  |  |  |  |
| Anger | 0.42 | 0.15 | .25 | 2.88 | .005 |  |  |  |  |  |
| Disgust | 0.41 | 0.13 | .25 | 3.04 | .003 |  |  |  |  |  |
| **Block 3** |  |  |  |  |  | 9.31 (5,121) | < .001 |  | .006 | .34 |
| Constant | 2.71 | 0.60 |  | 4.50 | < .001 |  |  |  |  |  |
| Age | -0.02 | 0.01 | -.27 | 3.43 | .001 |  |  |  |  |  |
| Anxiety | -0.09 | 0.18 | -.05 | 0.48 | .63 |  |  |  |  |  |
| Anger | 0.39 | 0.15 | .23 | 2.58 | .011 |  |  |  |  |  |
| Disgust | 0.38 | 0.14 | .23 | 2.75 | .007 |  |  |  |  |  |
| Depression | 0.21 | 0.22 | .11 | 0.97 | .34 |  |  |  |  |  |

*Note.* Bootstrap results are based on 1,000 bootstrap samples. BCa 95% CI = 95% bias-corrected and accelerated bootstrap confidence intervals. df = degrees of freedom.

# Table A.8 - Hierarchical Regression Models for Predicting Misophonic Responses (Weighted Score) in Misophonia Group at Baseline and Follow-Up

|  | ***B* [BCa 95% CI]** | ***SE B*** | ***β*** | ***t*** | ***p*** | ***F* (df)** | ***p*** | ***R^2^*** | **Δ*R^2^*** | ***p*** |
| --- | --- | --- | --- | --- | --- | --- | --- | --- | --- | --- |
| **BASELINE** |  |  |  |  |  |  |  |  |  |  |
| **Block 1** |  |  |  |  |  | 21.64 (1,489) | < .001 | .04 |  |  |
| Constant | 25.41 | 1.14 |  | 22.28 | < .001 |  |  |  |  |  |
| Age | -0.11 | 0.02 | -.21 | 4.65 | < .001 |  |  |  |  |  |
| **Block 2** |  |  |  |  |  | 60.33 (4,486) | < .001 |  | .29 | < .001 |
| Constant | 1.55 | 1.92 |  | 0.81 | .42 |  |  |  |  |  |
| Age | -0.05 | 0.02 | -.09 | 2.37 | .018 |  |  |  |  |  |
| Anxiety | 1.74 | 0.45 | .16 | 3.85 | < .001 |  |  |  |  |  |
| Anger | 2.76 | 0.48 | .25 | 5.73 | < .001 |  |  |  |  |  |
| Disgust | 3.19 | 0.44 | .30 | 7.34 | < .001 |  |  |  |  |  |
| **Block 3** |  |  |  |  |  | 54.05 (5,485) | < .001 |  | .03 | < .001 |
| Constant | 0.21 | 1.90 |  | 0.11 | .91 |  |  |  |  |  |
| Age | -0.05 | 0.02 | -.09 | 2.31 | .022 |  |  |  |  |  |
| Anxiety | 0.20 | 0.57 | .02 | 0.35 | .73 |  |  |  |  |  |
| Anger | 2.41 | 0.48 | .22 | 5.04 | < .001 |  |  |  |  |  |
| Disgust | 2.85 | 0.43 | .27 | 6.57 | < .001 |  |  |  |  |  |
| Depression | 3.02 | 0.68 | .24 | 4.43 | < .001 |  |  |  |  |  |
| **FOLLOW-UP** |  |  |  |  |  |  |  |  |  |  |
| **Block 1** |  |  |  |  |  | 8.60 (1,125) | .004 | .06 |  |  |
| Constant | 25.56 | 2.26 |  | 11.31 | < .001 |  |  |  |  |  |
| Age | -0.13 | 0.05 | -.25 | 2.93 | .004 |  |  |  |  |  |
| **Block 2** |  |  |  |  |  | 10.80 (4,122) | < .001 |  | .20 | < .001 |
| Constant | 7.21 | 4.25 |  | 1.70 | .092 |  |  |  |  |  |
| Age | -0.11 | 0.04 | -.21 | 2.69 | .008 |  |  |  |  |  |
| Anxiety | 0.21 | 0.99 | .02 | 0.21 | .84 |  |  |  |  |  |
| Anger | 3.75 | 1.05 | .32 | 3.57 | .001 |  |  |  |  |  |
| Disgust | 2.52 | 0.96 | .22 | 2.63 | .010 |  |  |  |  |  |
| **Block 3** |  |  |  |  |  | 8.95 (5,121) | < .001 |  | .01 | .24 |
| Constant | 6.30 | 4.31 |  | 1.46 | .15 |  |  |  |  |  |
| Age | -0.11 | 0.04 | -.20 | 2.53 | .013 |  |  |  |  |  |
| Anxiety | -0.75 | 1.27 | -.06 | 0.59 | .56 |  |  |  |  |  |
| Anger | 3.46 | 1.08 | .29 | 3.20 | .002 |  |  |  |  |  |
| Disgust | 2.26 | 0.98 | .20 | 2.32 | .022 |  |  |  |  |  |
| Depression | 1.88 | 1.58 | .14 | 1.19 | .24 |  |  |  |  |  |

*Note.* Bootstrap results are based on 1,000 bootstrap samples. BCa 95% CI = 95% bias-corrected and accelerated bootstrap confidence intervals. df = degrees of freedom.

# Table A.9 - Hierarchical Regression Models for Predicting QoL (Physical Functioning) in Misophonia Group at Baseline and Follow-Up

|  | ***B* [BCa 95% CI]** | ***SE B*** | ***β*** | ***t*** | ***p*** | ***F* (df)** | ***p*** | ***R^2^*** | **Δ*R^2^*** | ***p*** |
| --- | --- | --- | --- | --- | --- | --- | --- | --- | --- | --- |
| **BASELINE** |  |  |  |  |  |  |  |  |  |  |
| **Block 1** |  |  |  |  |  | 2.14 (1,489) | .14 | .004 |  |  |
| Constant | 88.89 | 2.96 |  | 30.00 | < .001 |  |  |  |  |  |
| Age | -0.09 | 0.06 | -.07 | 1.46 | .14 |  |  |  |  |  |
| **Block 2** |  |  |  |  |  | 6.98 (7,483) | < .001 |  | .09 | < .001 |
| Constant | 111.03 | 6.46 |  | 17.19 | < .001 |  |  |  |  |  |
| Age | -0.17 | 0.06 | -.12 | 2.56 | .01 |  |  |  |  |  |
| Frequency | -2.30 | 0.82 | -.15 | 2.83 | .005 |  |  |  |  |  |
| Recovery | -0.47 | 0.76 | -.03 | 0.62 | .54 |  |  |  |  |  |
| Avoidance | -1.28 | 0.64 | -.10 | 1.99 | .048 |  |  |  |  |  |
| Emotional | 1.72 | 1.00 | .10 | 1.73 | .084 |  |  |  |  |  |
| Physiological | -2.43 | 0.83 | -.16 | 2.94 | .003 |  |  |  |  |  |
| Participation | -0.66 | 0.86 | -.05 | 0.76 | .45 |  |  |  |  |  |
| **Block 3** |  |  |  |  |  | 8.21 (10,480) | < .001 |  | .05 | < .001 |
| Constant | 115.07 | 10.49 |  | 10.97 | < .001 |  |  |  |  |  |
| Age | -0.18 | 0.06 | -.13 | 2.91 | .004 |  |  |  |  |  |
| Frequency | -1.83 | 0.80 | -.12 | 2.30 | .022 |  |  |  |  |  |
| Recovery | 0.05 | 0.75 | .004 | 0.07 | .94 |  |  |  |  |  |
| Avoidance | -1.41 | 0.63 | -.11 | 2.22 | .027 |  |  |  |  |  |
| Emotional | 1.90 | 0.97 | .11 | 1.96 | .051 |  |  |  |  |  |
| Physiological | -1.94 | 0.81 | -.13 | 2.39 | .017 |  |  |  |  |  |
| Participation | 0.28 | 0.85 | .02 | 0.33 | .74 |  |  |  |  |  |
| Self-esteem | 1.46 | 1.93 | .05 | 0.76 | .45 |  |  |  |  |  |
| Anxiety | 0.39 | 1.71 | .01 | 0.23 | .82 |  |  |  |  |  |
| Depression | -7.89 | 2.24 | -.24 | 3.52 | < .001 |  |  |  |  |  |
| **FOLLOW-UP** |  |  |  |  |  |  |  |  |  |  |
| **Block 1** |  |  |  |  |  | 2.33 (1,125) | .13 | .02 |  |  |
| Constant | 93.14 | 5.67 |  | 16.42 | < .001 |  |  |  |  |  |
| Age | -0.17 | 0.11 | -.14 | 1.53 | .13 |  |  |  |  |  |
| **Block 2** |  |  |  |  |  | 2.31 (7,119) | .031 |  | .10 | .041 |
| Constant | 119.17 | 12.63 |  | 9.43 | < .001 |  |  |  |  |  |
| Age | -0.28 | 0.12 | -.21 | 2.34 | .021 |  |  |  |  |  |
| Frequency | -3.17 | 1.63 | -.20 | 1.95 | .05 |  |  |  |  |  |
| Recovery | -2.03 | 1.49 | -.14 | 1.37 | .17 |  |  |  |  |  |
| Avoidance | 0.66 | 1.41 | .05 | 0.47 | .64 |  |  |  |  |  |
| Emotional | 0.80 | 2.11 | .04 | 0.38 | .70 |  |  |  |  |  |
| Physiological | -1.85 | 1.54 | -.13 | 1.20 | .23 |  |  |  |  |  |
| Participation | -0.59 | 1.75 | -.04 | 0.34 | .74 |  |  |  |  |  |
| **Block 3** |  |  |  |  |  | 2.32 (10,116) | .016 |  | .05 | .093 |
| Constant | 132.16 | 24.70 |  | 5.35 | < .001 |  |  |  |  |  |
| Age | -0.30 | 0.12 | -.23 | 2.54 | .012 |  |  |  |  |  |
| Frequency | -2.60 | 1.62 | -.16 | 1.61 | .11 |  |  |  |  |  |
| Recovery | -1.09 | 1.52 | -.07 | 0.72 | .48 |  |  |  |  |  |
| Avoidance | 0.11 | 1.44 | .01 | 0.07 | .94 |  |  |  |  |  |
| Emotional | 1.20 | 2.18 | .06 | 0.55 | .58 |  |  |  |  |  |
| Physiological | -1.44 | 1.54 | -.10 | 0.94 | .35 |  |  |  |  |  |
| Participation | -0.06 | 1.76 | < -.01 | 0.03 | .97 |  |  |  |  |  |
| Self-esteem | -0.21 | 3.94 | -.01 | 0.05 | .96 |  |  |  |  |  |
| Anxiety | -1.26 | 3.47 | -.04 | 0.36 | .72 |  |  |  |  |  |
| Depression | -7.43 | 4.78 | -.22 | 1.55 | .12 |  |  |  |  |  |

*Note.* Bootstrap results are based on 1,000 bootstrap samples. BCa 95% CI = 95% bias-corrected and accelerated bootstrap confidence intervals. df = degrees of freedom.

# Table A.10 - Hierarchical Regression Models for Predicting QoL (Role Limitations – Physical Health) in Misophonia Group at Baseline and Follow-Up

|  | ***B* [BCa 95% CI]** | ***SE B*** | ***β*** | ***t*** | ***p*** | ***F* (df)** | ***p*** | ***R^2^*** | **Δ*R^2^*** | ***p*** |
| --- | --- | --- | --- | --- | --- | --- | --- | --- | --- | --- |
| **BASELINE** |  |  |  |  |  |  |  |  |  |  |
| **Block 1** |  |  |  |  |  | 3.39 (1,489) | .066 | .007 |  |  |
| Constant | 83.16 | 5.48 |  | 15.18 | < .001 |  |  |  |  |  |
| Age | -0.22 | 0.12 | -.08 | 1.84 | .066 |  |  |  |  |  |
| **Block 2** |  |  |  |  |  | 6.99 (7, 483) | < .001 |  | .09 | < .001 |
| Constant | 117.92 | 11.96 |  | 9.86 | < .001 |  |  |  |  |  |
| Age | -0.32 | 0.12 | -.12 | 2.68 | .008 |  |  |  |  |  |
| Frequency | -3.36 | 1.51 | -.11 | 2.22 | .027 |  |  |  |  |  |
| Recovery | -3.97 | 1.41 | -.14 | 2.81 | .005 |  |  |  |  |  |
| Avoidance | -1.78 | 1.19 | -.08 | 1.50 | .14 |  |  |  |  |  |
| Emotional | 4.53 | 1.84 | .14 | 2.46 | .014 |  |  |  |  |  |
| Physiological | -3.02 | 1.53 | -.11 | 1.97 | .049 |  |  |  |  |  |
| Participation | -1.62 | 1.59 | -.06 | 1.02 | .31 |  |  |  |  |  |
| **Block 3** |  |  |  |  |  | 12.61 (10, 480) | < .001 |  | .12 | < .001 |
| Constant | 149.18 | 18.71 |  | 7.97 | < .001 |  |  |  |  |  |
| Age | -0.35 | 0.11 | -.14 | 3.12 | .002 |  |  |  |  |  |
| Frequency | -2.11 | 1.42 | -.07 | 1.49 | .14 |  |  |  |  |  |
| Recovery | -2.66 | 1.34 | -.09 | 1.98 | .048 |  |  |  |  |  |
| Avoidance | -1.93 | 1.13 | -.08 | 1.71 | .088 |  |  |  |  |  |
| Emotional | 4.92 | 1.73 | .15 | 2.84 | .005 |  |  |  |  |  |
| Physiological | -1.61 | 1.45 | -.06 | 1.11 | .27 |  |  |  |  |  |
| Participation | 0.80 | 1.52 | .03 | 0.52 | .60 |  |  |  |  |  |
| Self-esteem | -0.91 | 3.44 | -.02 | 0.27 | .79 |  |  |  |  |  |
| Anxiety | 1.30 | 3.06 | -.03 | 0.42 | .67 |  |  |  |  |  |
| Depression | -24.94 | 4.00 | -.42 | 6.24 | < .001 |  |  |  |  |  |
| **FOLLOW-UP** |  |  |  |  |  |  |  |  |  |  |
| **Block 1** |  |  |  |  |  | 0.22 (1,125) | .64 | .002 |  |  |
| Constant | 80.92 | 10.59 |  | 7.64 | < .001 |  |  |  |  |  |
| Age | -0.10 | 0.21 | -.04 | 0.47 | .64 |  |  |  |  |  |
| **Block 2** |  |  |  |  |  | 1.24 (7,119) | .29 |  | .07 | .22 |
| Constant | 108.89 | 24.07 |  | 4.52 | < .001 |  |  |  |  |  |
| Age | -0.21 | 0.23 | -.09 | 0.92 | .36 |  |  |  |  |  |
| Frequency | -3.01 | 3.10 | -.10 | 0.97 | .33 |  |  |  |  |  |
| Recovery | -5.10 | 2.84 | -.19 | 1.80 | .074 |  |  |  |  |  |
| Avoidance | 1.87 | 2.68 | .08 | 0.70 | .49 |  |  |  |  |  |
| Emotional | 1.88 | 4.03 | .05 | 0.47 | .64 |  |  |  |  |  |
| Physiological | 0.95 | 2.93 | .04 | 0.32 | .75 |  |  |  |  |  |
| Participation | -3.48 | 3.33 | -.13 | 1.04 | .30 |  |  |  |  |  |
| **Block 3** |  |  |  |  |  | 2.22 (10,116) | .021 |  | .09 | .007 |
| Constant | 197.21 | 45.90 |  | 4.30 | < .001 |  |  |  |  |  |
| Age | -0.28 | 0.22 | -.12 | 1.30 | .20 |  |  |  |  |  |
| Frequency | -1.50 | 3.01 | -.05 | 0.50 | .62 |  |  |  |  |  |
| Recovery | -3.12 | 2.82 | -.12 | 1.11 | .27 |  |  |  |  |  |
| Avoidance | 1.45 | 2.68 | .06 | 0.54 | .59 |  |  |  |  |  |
| Emotional | 1.12 | 4.06 | .03 | 0.28 | .78 |  |  |  |  |  |
| Physiological | 2.56 | 2.87 | .10 | 0.89 | .38 |  |  |  |  |  |
| Participation | -2.99 | 3.27 | -.11 | 0.92 | .36 |  |  |  |  |  |
| Self-esteem | -11.43 | 7.31 | -.21 | 1.56 | .12 |  |  |  |  |  |
| Anxiety | -6.11 | 6.45 | -.12 | 0.95 | .35 |  |  |  |  |  |
| Depression | -22.94 | 8.89 | -.37 | 2.58 | .011 |  |  |  |  |  |

*Note.* Bootstrap results are based on 1,000 bootstrap samples. BCa 95% CI = 95% bias-corrected and accelerated bootstrap confidence intervals. df = degrees of freedom.

# Table A.11 - Hierarchical Regression Models for Predicting QoL (Role Limitations – Emotional Problems) in Misophonia Group at Baseline and Follow-Up

|  | ***B* [BCa 95% CI]** | ***SE B*** | ***β*** | ***t*** | ***p*** | ***F* (df)** | ***p*** | ***R^2^*** | **Δ*R^2^*** | ***p*** |
| --- | --- | --- | --- | --- | --- | --- | --- | --- | --- | --- |
| **BASELINE** |  |  |  |  |  |  |  |  |  |  |
| **Block 1** |  |  |  |  |  | 21.36 (1, 489) | < .001 | .04 |  |  |
| Constant | 22.83 | 6.16 |  | 3.70 | < .001 |  |  |  |  |  |
| Age | 0.61 | 0.13 | .21 | 4.62 | < .001 |  |  |  |  |  |
| **Block 2** |  |  |  |  |  | 11.29 (7, 483) | < .001 |  | .10 | < .001 |
| Constant | 86.45 | 13.33 |  | 6.49 | < .001 |  |  |  |  |  |
| Age | 0.40 | 0.13 | .13 | 3.00 | .003 |  |  |  |  |  |
| Frequency | -1.64 | 1.68 | -.05 | 0.97 | .33 |  |  |  |  |  |
| Recovery | -3.41 | 1.58 | -.11 | 2.16 | .031 |  |  |  |  |  |
| Avoidance | 1.73 | 1.33 | .07 | 1.30 | .19 |  |  |  |  |  |
| Emotional | -2.49 | 2.05 | -.07 | 1.21 | .23 |  |  |  |  |  |
| Physiological | -2.20 | 1.71 | -.07 | 1.29 | .20 |  |  |  |  |  |
| Participation | -5.31 | 1.77 | -.18 | 3.00 | .003 |  |  |  |  |  |
| **Block 3** |  |  |  |  |  | 28.98 (10, 480) | < .001 |  | .24 | < .001 |
| Constant | 103.32 | 19.01 |  | 5.44 | < .001 |  |  |  |  |  |
| Age | 0.31 | 0.11 | .11 | 2.73 | .007 |  |  |  |  |  |
| Frequency | 0.45 | 1.45 | .01 | 0.31 | .76 |  |  |  |  |  |
| Recovery | -1.02 | 1.36 | -.03 | 0.75 | .45 |  |  |  |  |  |
| Avoidance | 1.08 | 1.15 | .04 | 0.95 | .35 |  |  |  |  |  |
| Emotional | -1.69 | 1.76 | -.04 | 0.96 | .34 |  |  |  |  |  |
| Physiological | -0.01 | 1.47 | < .01 | 0.01 | .99 |  |  |  |  |  |
| Participation | -1.14 | 1.55 | -.04 | 0.74 | .46 |  |  |  |  |  |
| Self-esteem | 6.83 | 3.50 | .10 | 1.95 | .051 |  |  |  |  |  |
| Anxiety | 0.55 | 3.11 | .01 | 0.18 | .86 |  |  |  |  |  |
| Depression | -33.69 | 4.06 | -.49 | 8.30 | < .001 |  |  |  |  |  |
| **FOLLOW-UP** |  |  |  |  |  |  |  |  |  |  |
| **Block 1** |  |  |  |  |  | 10.74 (1,125) | .001 | .08 |  |  |
| Constant | 15.20 | 11.93 |  | 1.27 | .21 |  |  |  |  |  |
| Age | 0.79 | 0.24 | .28 | 3.28 | .001 |  |  |  |  |  |
| **Block 2** |  |  |  |  |  | 4.33 (7,119) | < .001 |  | .12 | .008 |
| Constant | 75.09 | 26.11 |  | 2.88 | .005 |  |  |  |  |  |
| Age | 0.51 | 0.24 | .18 | 2.09 | .039 |  |  |  |  |  |
| Frequency | 3.23 | 3.36 | .09 | 0.97 | .34 |  |  |  |  |  |
| Recovery | -1.70 | 3.08 | -.05 | 0.55 | .58 |  |  |  |  |  |
| Avoidance | 8.97 | 2.90 | .34 | 3.09 | .002 |  |  |  |  |  |
| Emotional | -12.53 | 4.37 | -.30 | 2.87 | .005 |  |  |  |  |  |
| Physiological | 1.47 | 3.18 | .05 | 0.46 | .65 |  |  |  |  |  |
| Participation | -8.94 | 3.62 | -.29 | 2.47 | .015 |  |  |  |  |  |
| **Block 3** |  |  |  |  |  | 8.00 (10,116) | < .001 |  | .21 | < .001 |
| Constant | 170.19 | 45.21 |  | 3.76 | < .001 |  |  |  |  |  |
| Age | 0.39 | 0.21 | .14 | 1.82 | .071 |  |  |  |  |  |
| Frequency | 5.80 | 2.96 | .16 | 1.96 | .053 |  |  |  |  |  |
| Recovery | 2.46 | 2.78 | .08 | 0.89 | .38 |  |  |  |  |  |
| Avoidance | 7.43 | 2.64 | .28 | 2.81 | .006 |  |  |  |  |  |
| Emotional | -12.32 | 4.00 | -.30 | 3.08 | .003 |  |  |  |  |  |
| Physiological | 3.55 | 2.83 | .12 | 1.26 | .21 |  |  |  |  |  |
| Participation | -7.03 | 3.22 | -.23 | 2.18 | .031 |  |  |  |  |  |
| Self-esteem | -8.40 | 7.20 | -.13 | 1.17 | .25 |  |  |  |  |  |
| Anxiety | 0.47 | 6.35 | .01 | 0.07 | .94 |  |  |  |  |  |
| Depression | -43.71 | 8.75 | -.60 | 4.99 | < .001 |  |  |  |  |  |

*Note.* Bootstrap results are based on 1,000 bootstrap samples. BCa 95% CI = 95% bias-corrected and accelerated bootstrap confidence intervals. df = degrees of freedom.

# Table A.12 - Hierarchical Regression Models for Predicting QoL (Energy/Fatigue) in Misophonia Group at Baseline and Follow-Up

|  | ***B* [BCa 95% CI]** | ***SE B*** | ***β*** | ***t*** | ***p*** | ***F* (df)** | ***p*** | ***R^2^*** | **Δ*R^2^*** | ***p*** |
| --- | --- | --- | --- | --- | --- | --- | --- | --- | --- | --- |
| **BASELINE** |  |  |  |  |  |  |  |  |  |  |
| **Block 1** |  |  |  |  |  | 11.31 (1,489) | .001 | .02 |  |  |
| Constant | 31.59 | 3.09 |  | 10.22 | < .001 |  |  |  |  |  |
| Age | 0.22 | 0.07 | .15 | 3.36 | .001 |  |  |  |  |  |
| **Block 2** |  |  |  |  |  | 12.37 (7,483) | < .001 |  | .13 | < .001 |
| Constant | 66.75 | 6.58 |  | 10.15 | < .001 |  |  |  |  |  |
| Age | 0.09 | 0.07 | .06 | 1.41 | .16 |  |  |  |  |  |
| Frequency | -1.62 | 0.83 | -.10 | 1.95 | .052 |  |  |  |  |  |
| Recovery | -1.95 | 0.78 | -.12 | 2.51 | .012 |  |  |  |  |  |
| Avoidance | 1.07 | 0.65 | .08 | 1.63 | .10 |  |  |  |  |  |
| Emotional | -0.84 | 1.01 | -.04 | 0.83 | .41 |  |  |  |  |  |
| Physiological | -2.01 | 0.84 | -.13 | 2.38 | .018 |  |  |  |  |  |
| Participation | -2.31 | 0.87 | -.16 | 2.64 | .009 |  |  |  |  |  |
| **Block 3** |  |  |  |  |  | 49.49 (10,480) | < .001 |  | .36 | < .001 |
| Constant | 75.20 | 8.39 |  | 8.96 | < .001 |  |  |  |  |  |
| Age | 0.04 | 0.05 | .03 | 0.76 | .45 |  |  |  |  |  |
| Frequency | -0.35 | 0.64 | -.02 | 0.54 | .59 |  |  |  |  |  |
| Recovery | -0.43 | 0.60 | -.03 | 0.71 | .48 |  |  |  |  |  |
| Avoidance | 0.57 | 0.51 | .04 | 1.13 | .26 |  |  |  |  |  |
| Emotional | -0.31 | 0.78 | -.02 | 0.41 | .69 |  |  |  |  |  |
| Physiological | -0.65 | 0.65 | -.04 | 0.99 | .32 |  |  |  |  |  |
| Participation | 0.27 | 0.68 | .02 | 0.40 | .69 |  |  |  |  |  |
| Self-esteem | 4.74 | 1.54 | .14 | 3.07 | .002 |  |  |  |  |  |
| Anxiety | -1.42 | 1.37 | -.05 | 1.03 | .30 |  |  |  |  |  |
| Depression | -18.64 | 1.79 | -.55 | 10.41 | < .001 |  |  |  |  |  |
| **FOLLOW-UP** |  |  |  |  |  |  |  |  |  |  |
| **Block 1** |  |  |  |  |  | 8.88 (1,125) | .003 | .07 |  |  |
| Constant | 24.97 | 6.00 |  | 4.16 | < .001 |  |  |  |  |  |
| Age | 0.36 | 0.12 | .26 | 2.98 | .003 |  |  |  |  |  |
| **Block 2** |  |  |  |  |  | 3.73 (7,119) | .001 |  | .11 | .015 |
| Constant | 67.04 | 13.22 |  | 5.07 | < .001 |  |  |  |  |  |
| Age | 0.26 | 0.12 | .19 | 2.12 | .036 |  |  |  |  |  |
| Frequency | -1.23 | 1.70 | -.07 | 0.72 | .47 |  |  |  |  |  |
| Recovery | -2.35 | 1.56 | -.15 | 1.51 | .13 |  |  |  |  |  |
| Avoidance | 1.41 | 1.47 | .11 | 0.96 | .34 |  |  |  |  |  |
| Emotional | -4.50 | 2.21 | -.22 | 2.03 | .044 |  |  |  |  |  |
| Physiological | 1.84 | 1.61 | .12 | 1.14 | .26 |  |  |  |  |  |
| Participation | -2.68 | 1.83 | -.17 | 1.46 | .15 |  |  |  |  |  |
| **Block 3** |  |  |  |  |  | 5.65 (10,116) | < .001 |  | .15 | < .001 |
| Constant | 85.91 | 24.06 |  | 3.57 | .001 |  |  |  |  |  |
| Age | 0.22 | 0.11 | .16 | 1.96 | .052 |  |  |  |  |  |
| Frequency | -0.15 | 1.58 | -.01 | 0.09 | .93 |  |  |  |  |  |
| Recovery | -0.51 | 1.48 | -.03 | 0.34 | .73 |  |  |  |  |  |
| Avoidance | 0.27 | 1.41 | .02 | 0.19 | .85 |  |  |  |  |  |
| Emotional | -3.56 | 2.13 | -.17 | 1.67 | .097 |  |  |  |  |  |
| Physiological | 2.54 | 1.50 | .17 | 1.69 | .093 |  |  |  |  |  |
| Participation | -1.58 | 1.71 | -.10 | 0.92 | .36 |  |  |  |  |  |
| Self-esteem | 0.74 | 3.83 | .02 | 0.19 | .85 |  |  |  |  |  |
| Anxiety | -2.12 | 3.38 | -.07 | 0.63 | .53 |  |  |  |  |  |
| Depression | -13.63 | 4.66 | -.38 | 2.93 | .004 |  |  |  |  |  |

*Note.* Bootstrap results are based on 1,000 bootstrap samples. BCa 95% CI = 95% bias-corrected and accelerated bootstrap confidence intervals. df = degrees of freedom.

# Table A.13 - Hierarchical Regression Models for Predicting QoL (Emotional Wellbeing) in Misophonia Group at Baseline and Follow-Up

|  | ***B* [BCa 95% CI]** | ***SE B*** | ***β*** | ***t*** | ***p*** | ***F* (df)** | ***p*** | ***R^2^*** | **Δ*R^2^*** | ***p*** |
| --- | --- | --- | --- | --- | --- | --- | --- | --- | --- | --- |
| **BASELINE** |  |  |  |  |  |  |  |  |  |  |
| **Block 1** |  |  |  |  |  | 14.68 (1,489) | < .001 | .03 |  |  |
| Constant | 45.12 | 2.87 |  | 15.73 | < .001 |  |  |  |  |  |
| Age | 0.24 | 0.06 | .17 | 3.83 | < .001 |  |  |  |  |  |
| **Block 2** |  |  |  |  |  | 18.44 (7,483) | < .001 |  | .18 | < .001 |
| Constant | 86.33 | 5.90 |  | 14.62 | < .001 |  |  |  |  |  |
| Age | 0.10 | 0.06 | .07 | 1.70 | .090 |  |  |  |  |  |
| Frequency | -1.24 | 0.75 | -.08 | 1.66 | .097 |  |  |  |  |  |
| Recovery | -2.06 | 0.70 | -.14 | 2.95 | .003 |  |  |  |  |  |
| Avoidance | 0.85 | 0.69 | .07 | 1.44 | .15 |  |  |  |  |  |
| Emotional | -1.60 | 0.91 | -.09 | 1.76 | .079 |  |  |  |  |  |
| Physiological | -1.43 | 0.76 | -.10 | 1.89 | .059 |  |  |  |  |  |
| Participation | -3.10 | 0.79 | -.23 | 3.95 | < .001 |  |  |  |  |  |
| **Block 3** |  |  |  |  |  | 125.75 (10,480) | < .001 |  | .51 | < .001 |
| Constant | 94.26 | 5.85 |  | 16.11 | < .001 |  |  |  |  |  |
| Age | 0.04 | 0.04 | .03 | 1.13 | .26 |  |  |  |  |  |
| Frequency | 0.14 | 0.45 | .009 | 0.31 | .76 |  |  |  |  |  |
| Recovery | -0.18 | 0.42 | -.01 | 0.43 | .67 |  |  |  |  |  |
| Avoidance | 0.02 | 0.35 | < .01 | 0.04 | .97 |  |  |  |  |  |
| Emotional | -0.92 | 0.54 | -.05 | 1.69 | .091 |  |  |  |  |  |
| Physiological | 0.15 | 0.45 | .01 | 0.34 | .74 |  |  |  |  |  |
| Participation | -0.21 | 0.48 | -.02 | 0.44 | .66 |  |  |  |  |  |
| Self-esteem | 5.89 | 1.08 | .19 | 5.47 | < .001 |  |  |  |  |  |
| Anxiety | -0.72 | 0.96 | -.27 | 7.58 | < .001 |  |  |  |  |  |
| Depression | -14.85 | 1.25 | -.47 | 11.89 | < .001 |  |  |  |  |  |
| **FOLLOW-UP** |  |  |  |  |  |  |  |  |  |  |
| **Block 1** |  |  |  |  |  | 9.25 (1,125) | .003 | .07 |  |  |
| Constant | 40.83 | 5.72 |  | 7.14 | < .001 |  |  |  |  |  |
| Age | 0.35 | 0.12 | .26 | 3.04 | .003 |  |  |  |  |  |
| **Block 2** |  |  |  |  |  | 3.97 (7,119) | .001 |  | .12 | .010 |
| Constant | 78.07 | 12.55 |  | 6.22 | < .001 |  |  |  |  |  |
| Age | 0.23 | 0.18 | .17 | 1.97 | .051 |  |  |  |  |  |
| Frequency | -2.49 | 1.62 | -.15 | 1.54 | .13 |  |  |  |  |  |
| Recovery | -2.33 | 1.48 | -.15 | 1.58 | .12 |  |  |  |  |  |
| Avoidance | 1.74 | 1.40 | .14 | 1.25 | .22 |  |  |  |  |  |
| Emotional | -2.14 | 2.10 | -.11 | 1.02 | .31 |  |  |  |  |  |
| Physiological | 0.32 | 1.53 | .02 | 0.21 | .83 |  |  |  |  |  |
| Participation | -2.58 | 1.74 | -.18 | 1.48 | .14 |  |  |  |  |  |
| **Block 3** |  |  |  |  |  | 11.54 (10,116) | < .001 |  | .31 | < .001 |
| Constant | 79.17 | 19.84 |  | 3.99 | < .001 |  |  |  |  |  |
| Age | 0.20 | 0.09 | .15 | 2.09 | .039 |  |  |  |  |  |
| Frequency | -1.08 | 1.30 | -.06 | 0.83 | .41 |  |  |  |  |  |
| Recovery | 0.13 | 1.22 | .01 | 0.11 | .91 |  |  |  |  |  |
| Avoidance | -0.31 | 1.16 | -.02 | 0.26 | .79 |  |  |  |  |  |
| Emotional | 0.02 | 1.76 | < .01 | 0.01 | .99 |  |  |  |  |  |
| Physiological | 1.06 | 1.24 | .07 | 0.85 | .40 |  |  |  |  |  |
| Participation | -0.85 | 1.41 | -.06 | 0.60 | .55 |  |  |  |  |  |
| Self-esteem | 5.65 | 3.16 | .18 | 1.79 | .077 |  |  |  |  |  |
| Anxiety | -5.73 | 2.79 | -.19 | 2.05 | .042 |  |  |  |  |  |
| Depression | -11.90 | 3.84 | -.34 | 3.10 | .002 |  |  |  |  |  |

*Note.* Bootstrap results are based on 1,000 bootstrap samples. BCa 95% CI = 95% bias-corrected and accelerated bootstrap confidence intervals. df = degrees of freedom.

# Table A.14 - Hierarchical Regression Models for Predicting QoL (Social Functioning) in Misophonia Group at Baseline and Follow-Up

|  | ***B* [BCa 95% CI]** | ***SE B*** | ***β*** | ***t*** | ***p*** | ***F* (df)** | ***p*** | ***R^2^*** | **Δ*R^2^*** | ***p*** |
| --- | --- | --- | --- | --- | --- | --- | --- | --- | --- | --- |
| **BASELINE** |  |  |  |  |  |  |  |  |  |  |
| **Block 1** |  |  |  |  |  | 11.17 (1,489) | .001 | .02 |  |  |
| Constant | 48.43 | 4.05 |  | 11.95 | < .001 |  |  |  |  |  |
| Age | 0.29 | 0.09 | .15 | 3.34 | .001 |  |  |  |  |  |
| **Block 2** |  |  |  |  |  | 28.77 (7,483) | < .001 |  | .27 | < .001 |
| Constant | 111.36 | 7.86 |  | 15.16 | < .001 |  |  |  |  |  |
| Age | 0.09 | 0.08 | .04 | 1.10 | .27 |  |  |  |  |  |
| Frequency | -3.07 | 0.99 | -.14 | 3.09 | .002 |  |  |  |  |  |
| Recovery | -3.05 | 0.93 | -.14 | 3.28 | .001 |  |  |  |  |  |
| Avoidance | 0.26 | 0.78 | .02 | 0.34 | .74 |  |  |  |  |  |
| Emotional | 0.46 | 1.21 | .02 | 0.38 | .71 |  |  |  |  |  |
| Physiological | -3.13 | 1.01 | -.15 | 3.10 | .002 |  |  |  |  |  |
| Participation | -5.57 | 1.05 | -.29 | 5.33 | < .001 |  |  |  |  |  |
| **Block 3** |  |  |  |  |  | 45.63 (10,480) | < .001 |  | .19 | < .001 |
| Constant | 131.58 | 11.22 |  | 11.72 | < .001 |  |  |  |  |  |
| Age | 0.05 | 0.07 | .02 | 0.67 | .51 |  |  |  |  |  |
| Frequency | -1.85 | 0.85 | -.08 | 2.17 | .031 |  |  |  |  |  |
| Recovery | -1.58 | 0.81 | -.08 | 1.96 | .051 |  |  |  |  |  |
| Avoidance | -0.17 | 0.68 | -.01 | 0.25 | .80 |  |  |  |  |  |
| Emotional | 0.95 | 1.04 | .04 | 0.92 | .36 |  |  |  |  |  |
| Physiological | -1.73 | 0.87 | -.08 | 1.98 | .048 |  |  |  |  |  |
| Participation | -3.11 | 0.91 | -.16 | 3.41 | .001 |  |  |  |  |  |
| Self-esteem | 1.85 | 2.07 | -.04 | 0.89 | .37 |  |  |  |  |  |
| Anxiety | -2.59 | 1.83 | -.07 | 1.42 | .16 |  |  |  |  |  |
| Depression | -19.05 | 2.40 | -.43 | 7.95 | < .001 |  |  |  |  |  |
| **FOLLOW-UP** |  |  |  |  |  |  |  |  |  |  |
| **Block 1** |  |  |  |  |  | 1.99 (1,125) | .16 | .02 |  |  |
| Constant | 56.61 | 8.26 |  | 6.85 | < .001 |  |  |  |  |  |
| Age | 0.23 | 0.17 | .13 | 1.41 | .16 |  |  |  |  |  |
| **Block 2** |  |  |  |  |  | 2.50 (7,119) | .020 |  | .11 | .023 |
| Constant | 104.29 | 18.28 |  | 5.70 | < .001 |  |  |  |  |  |
| Age | 0.10 | 0.17 | .05 | 0.56 | .58 |  |  |  |  |  |
| Frequency | -1.98 | 2.35 | -.08 | 0.84 | .40 |  |  |  |  |  |
| Recovery | -3.50 | 2.15 | -.16 | 1.62 | .11 |  |  |  |  |  |
| Avoidance | 0.38 | 2.03 | .02 | 0.19 | .85 |  |  |  |  |  |
| Emotional | -0.82 | 3.06 | -.03 | 0.27 | .79 |  |  |  |  |  |
| Physiological | -1.79 | 2.23 | -.09 | 0.80 | .42 |  |  |  |  |  |
| Participation | -2.57 | 2.53 | -.13 | 1.02 | .31 |  |  |  |  |  |
| **Block 3** |  |  |  |  |  | 3.51 (10,116) | < .001 |  | .10 | .002 |
| Constant | 140.90 | 34.48 |  | 4.09 | < .001 |  |  |  |  |  |
| Age | 0.05 | 0.16 | .03 | 0.30 | .76 |  |  |  |  |  |
| Frequency | -0.71 | 2.26 | -.03 | 0.32 | .75 |  |  |  |  |  |
| Recovery | -1.57 | 2.12 | -.07 | 00.74 | .46 |  |  |  |  |  |
| Avoidance | -0.76 | 2.02 | -.04 | 0.38 | .71 |  |  |  |  |  |
| Emotional | -0.10 | 3.05 | < -.01 | 0.03 | .97 |  |  |  |  |  |
| Physiological | -0.78 | 2.15 | -.04 | 0.36 | .72 |  |  |  |  |  |
| Participation | -1.60 | 2.45 | -.08 | 0.65 | .52 |  |  |  |  |  |
| Self-esteem | -2.06 | 5.49 | -.05 | 0.38 | .71 |  |  |  |  |  |
| Anxiety | -6.10 | 4.85 | -.15 | 1.26 | .21 |  |  |  |  |  |
| Depression | -13.90 | 6.68 | -.29 | 2.08 | .040 |  |  |  |  |  |

*Note.* Bootstrap results are based on 1,000 bootstrap samples. BCa 95% CI = 95% bias-corrected and accelerated bootstrap confidence intervals. df = degrees of freedom.

# Table A.15 - Hierarchical Regression Models for Predicting QoL (Pain) in Misophonia Group at Baseline and Follow-Up

|  | ***B* [BCa 95% CI]** | ***SE B*** | ***β*** | ***t*** | ***p*** | ***F* (df)** | ***p*** | ***R^2^*** | **Δ*R^2^*** | ***p*** |
| --- | --- | --- | --- | --- | --- | --- | --- | --- | --- | --- |
| **BASELINE** |  |  |  |  |  |  |  |  |  |  |
| **Block 1** |  |  |  |  |  | 2.42 (1,489) | .12 | .005 |  |  |
| Constant | 75.66 | 3.55 |  | 21.33 | < .001 |  |  |  |  |  |
| Age | -0.12 | 0.08 | -.07 | 1.56 | .12 |  |  |  |  |  |
| **Block 2** |  |  |  |  |  | 9.72 (7,483) | < .001 |  | .12 | < .001 |
| Constant | 100.86 | 7.60 |  | 13.27 | < .001 |  |  |  |  |  |
| Age | -0.21 | 0.08 | -.13 | 2.78 | .006 |  |  |  |  |  |
| Frequency | -2.55 | 0.96 | -.13 | 2.66 | .008 |  |  |  |  |  |
| Recovery | -2.43 | 0.90 | -.13 | 2.70 | .007 |  |  |  |  |  |
| Avoidance | -0.80 | 0.76 | -.05 | 1.06 | .29 |  |  |  |  |  |
| Emotional | 3.39 | 1.17 | .16 | 2.90 | .004 |  |  |  |  |  |
| Physiological | -3.28 | 0.97 | -.18 | 3.37 | .001 |  |  |  |  |  |
| Participation | -1.26 | 1.01 | -.08 | 1.25 | .21 |  |  |  |  |  |
| **Block 3** |  |  |  |  |  | 13.49 (10,480) | < .001 |  | .10 | < .001 |
| Constant | 114.72 | 12.01 |  | 9.55 | < .001 |  |  |  |  |  |
| Age | -0.23 | 0.07 | -.14 | 3.23 | .001 |  |  |  |  |  |
| Frequency | -1.81 | 0.91 | -.10 | 1.98 | .048 |  |  |  |  |  |
| Recovery | -1.61 | 0.86 | -.09 | 1.87 | .063 |  |  |  |  |  |
| Avoidance | -0.96 | 0.73 | -.06 | 1.32 | .19 |  |  |  |  |  |
| Emotional | 3.65 | 1.11 | .17 | 3.29 | .001 |  |  |  |  |  |
| Physiological | -2.46 | 0.93 | .14 | 2.64 | .009 |  |  |  |  |  |
| Participation | 0.21 | 0.98 | .01 | 0.21 | .83 |  |  |  |  |  |
| Self-esteem | 0.61 | 2.21 | .02 | 0.28 | .78 |  |  |  |  |  |
| Anxiety | 0.29 | 1.96 | .01 | 0.15 | .88 |  |  |  |  |  |
| Depression | -13.56 | 2.57 | -.35 | 5.29 | < .001 |  |  |  |  |  |
| **FOLLOW-UP** |  |  |  |  |  |  |  |  |  |  |
| **Block 1** |  |  |  |  |  | 0.35 (1,125) | .56 | .003 |  |  |
| Constant | 76.33 | 6.78 |  | 11.26 | < .001 |  |  |  |  |  |
| Age | -0.08 | 0.14 | -.05 | 0.59 | .56 |  |  |  |  |  |
| **Block 2** |  |  |  |  |  | 2.43 (7,119) | .023 |  | .12 | .015 |
| Constant | 90.40 | 14.93 |  | 6.06 | < .001 |  |  |  |  |  |
| Age | -0.22 | 0.14 | -.14 | 1.58 | .12 |  |  |  |  |  |
| Frequency | -0.47 | 1.92 | -.02 | 0.24 | .81 |  |  |  |  |  |
| Recovery | -1.00 | 1.76 | -.06 | 0.57 | .57 |  |  |  |  |  |
| Avoidance | 3.07 | 1.66 | .21 | 1.85 | .067 |  |  |  |  |  |
| Emotional | 2.03 | 2.50 | .09 | 0.81 | .42 |  |  |  |  |  |
| Physiological | -3.18 | 1.82 | -.19 | 1.75 | .083 |  |  |  |  |  |
| Participation | -4.91 | 2.07 | -.29 | 2.38 | .019 |  |  |  |  |  |
| **Block 3** |  |  |  |  |  | 2.39 (10,116) | .013 |  | .05 | .10 |
| Constant | 122.50 | 29.21 |  | 4.20 | < .001 |  |  |  |  |  |
| Age | -0.25 | 0.14 | -.17 | 1.82 | .071 |  |  |  |  |  |
| Frequency | 0.22 | 1.91 | .01 | 0.12 | .91 |  |  |  |  |  |
| Recovery | < - .01 | 1.79 | < .01 | < .01 | 1.00 |  |  |  |  |  |
| Avoidance | 2.74 | 1.71 | .19 | 1.60 | .11 |  |  |  |  |  |
| Emotional | 1.94 | 2.58 | .09 | 0.75 | .45 |  |  |  |  |  |
| Physiological | -2.53 | 1.83 | -.15 | 1.39 | .17 |  |  |  |  |  |
| Participation | -4.54 | 2.08 | -.27 | 2.19 | .031 |  |  |  |  |  |
| Self-esteem | -3.58 | 4.65 | -.10 | 0.77 | .44 |  |  |  |  |  |
| Anxiety | -1.96 | 4.11 | -.06 | 0.48 | .63 |  |  |  |  |  |
| Depression | -10.34 | 5.66 | -.26 | 1.83 | .070 |  |  |  |  |  |

*Note.* Bootstrap results are based on 1,000 bootstrap samples. BCa 95% CI = 95% bias-corrected and accelerated bootstrap confidence intervals. df = degrees of freedom.

# Table A.16 - Hierarchical Regression Models for Predicting QoL (General Health) in Misophonia Group at Baseline and Follow-Up

|  | ***B* [BCa 95% CI]** | ***SE B*** | ***β*** | ***t*** | ***p*** | ***F* (df)** | ***p*** | ***R^2^*** | **Δ*R^2^*** | ***p*** |
| --- | --- | --- | --- | --- | --- | --- | --- | --- | --- | --- |
| **BASELINE** |  |  |  |  |  |  |  |  |  |  |
| **Block 1** |  |  |  |  |  | 0.67 (1,489) | .41 | .001 |  |  |
| Constant | 57.44 | 3.20 |  | 17.94 | < .001 |  |  |  |  |  |
| Age | 0.06 | 0.07 | .04 | 0.82 | .41 |  |  |  |  |  |
| **Block 2** |  |  |  |  |  | 9.60 (7,483) | < .001 |  | .12 | < .001 |
| Constant | 79.82 | 6.85 |  | 11.65 | < .001 |  |  |  |  |  |
| Age | -0.04 | 0.07 | -.02 | 0.54 | .59 |  |  |  |  |  |
| Frequency | -2.05 | 0.87 | -.12 | 2.37 | .018 |  |  |  |  |  |
| Recovery | -2.36 | 0.81 | -.14 | 2.91 | .004 |  |  |  |  |  |
| Avoidance | 0.57 | 0.68 | .04 | 0.83 | .41 |  |  |  |  |  |
| Emotional | 2.44 | 1.06 | .13 | 2.31 | .021 |  |  |  |  |  |
| Physiological | -2.38 | 0.88 | -.15 | 2.71 | .007 |  |  |  |  |  |
| Participation | -2.33 | 0.91 | -.15 | 2.56 | .011 |  |  |  |  |  |
| **Block 3** |  |  |  |  |  | 18.66 (10,480) | < .001 |  | .16 | < .001 |
| Constant | 73.38 | 10.39 |  | 7.06 | < .001 |  |  |  |  |  |
| Age | -0.08 | 0.06 | -.06 | 1.34 | .18 |  |  |  |  |  |
| Frequency | -1.20 | 0.79 | -.07 | 1.51 | .13 |  |  |  |  |  |
| Recovery | -1.26 | 0.75 | -.08 | 1.69 | .092 |  |  |  |  |  |
| Avoidance | 0.07 | 0.63 | .01 | 0.12 | .92 |  |  |  |  |  |
| Emotional | 2.85 | 0.96 | .15 | 2.97 | .003 |  |  |  |  |  |
| Physiological | -1.51 | 0.81 | -.09 | 1.88 | .061 |  |  |  |  |  |
| Participation | -0.55 | 0.85 | -.04 | 0.65 | .52 |  |  |  |  |  |
| Self-esteem | 6.07 | 1.91 | .18 | 3.17 | .002 |  |  |  |  |  |
| Anxiety | -2.17 | 1.70 | -.07 | 1.28 | .20 |  |  |  |  |  |
| Depression | -9.10 | 2.22 | -.26 | 4.10 | < .001 |  |  |  |  |  |
| **FOLLOW-UP** |  |  |  |  |  |  |  |  |  |  |
| **Block 1** |  |  |  |  |  | 0.28 (1,125) | .60 | .002 |  |  |
| Constant | 60.70 | 5.86 |  | 10.36 | < .001 |  |  |  |  |  |
| Age | 0.06 | 0.12 | .05 | 0.53 | .60 |  |  |  |  |  |
| **Block 2** |  |  |  |  |  | 1.89 (7,119) | .078 |  | .10 | .053 |
| Constant | 72.27 | 13.09 |  | 5,52 | < .001 |  |  |  |  |  |
| Age | -0.02 | 0.12 | -.02 | 0.19 | .85 |  |  |  |  |  |
| Frequency | -2.90 | 1.68 | -.17 | 1.72 | .087 |  |  |  |  |  |
| Recovery | -2.22 | 1.54 | -.15 | 1.44 | .15 |  |  |  |  |  |
| Avoidance | 2.91 | 1.46 | .23 | 2.00 | .048 |  |  |  |  |  |
| Emotional | 0.76 | 2.19 | .04 | 0.35 | .73 |  |  |  |  |  |
| Physiological | 1.09 | 1.59 | .08 | 0.68 | .50 |  |  |  |  |  |
| Participation | -2.62 | 1.81 | -.18 | 1.45 | .15 |  |  |  |  |  |
| **Block 3** |  |  |  |  |  | 2.30 (10,116) | .017 |  | .07 | .032 |
| Constant | 83.58 | 25.32 |  | 3.30 | .001 |  |  |  |  |  |
| Age | -0.05 | 0.12 | -.04 | 0.39 | .70 |  |  |  |  |  |
| Frequency | -2.23 | 1.66 | -.13 | 1.34 | .18 |  |  |  |  |  |
| Recovery | -1.06 | 1.55 | -.07 | 0.68 | .50 |  |  |  |  |  |
| Avoidance | 2.19 | 1.48 | .18 | 1.48 | .14 |  |  |  |  |  |
| Emotional | 1.35 | 2.24 | .07 | 0.60 | .55 |  |  |  |  |  |
| Physiological | 1.52 | 1.58 | .11 | 0.96 | .34 |  |  |  |  |  |
| Participation | -1.92 | 1.80 | -.13 | 1.07 | .29 |  |  |  |  |  |
| Self-esteem | 0.56 | 4.03 | .02 | 0.14 | .89 |  |  |  |  |  |
| Anxiety | -1.02 | 3.56 | -.04 | 0.29 | .78 |  |  |  |  |  |
| Depression | -8.78 | 4.90 | -.26 | 1.79 | .076 |  |  |  |  |  |

*Note.* Bootstrap results are based on 1,000 bootstrap samples. BCa 95% CI = 95% bias-corrected and accelerated bootstrap confidence intervals. df = degrees of freedom.
